# Differences between winter oilseed rape (*Brassica napus* L.) cultivars in nitrogen starvation-induced leaf senescence are governed by leaf-inherent rather than root-derived signals

**DOI:** 10.1093/jxb/erv170

**Published:** 2015-05-04

**Authors:** Fabian Koeslin-Findeklee, Martin A. Becker, Eric van der Graaff, Thomas Roitsch, Walter J. Horst

**Affiliations:** ^1^Institute of Plant Nutrition, Leibniz University of Hannover, Herrenhäuser Str. 2, D-30419 Hannover, Germany; ^2^Department of Plant and Environmental Sciences, Copenhagen Plant Science Center, University of Copenhagen, Højbakkegård Allé13, DK-2630 Taastrup, Denmark; ^3^Global Change Research Centre, CzechGlobe AS CR, v.v.i., Drásov 470, Cz-664 24 Drásov, Czech Republic

**Keywords:** *Brassica napus*, cytokinins, genotypic differences, leaf senescence, nitrogen efficiency, nitrogen starvation, reciprocal grafting, stay-green.

## Abstract

Nitrogen (N) efficiency of winter oilseed rape (*Brassica napus* L.) line-cultivars (cvs.), defined as high grain yield under N limitation, has been primarily attributed to maintained N uptake during reproductive growth (N uptake efficiency) in combination with delayed senescence of the older leaves accompanied with maintained photosynthetic capacity (functional stay-green). However, it is not clear whether genotypic variation in N starvation-induced leaf senescence is due to leaf-inherent factors and/or governed by root-mediated signals. Therefore, the N-efficient and stay-green cvs. NPZ-1 and Apex were reciprocally grafted with the N-inefficient and early-senescing cvs. NPZ-2 and Capitol, respectively and grown in hydroponics. The senescence status of older leaves after 12 days of N starvation assessed by SPAD, photosynthesis and the expression of the senescence-specific cysteine protease gene *SAG12-1* revealed that the stay-green phenotype of the cvs. NPZ-1 and Apex under N starvation was primarily under the control of leaf-inherent factors. The same four cultivars were submitted to N starvation for up to 12 days in a time-course experiment. The specific leaf contents of biologically active and inactive cytokinins (CKs) and the expression of genes involved in CK homeostasis revealed that under N starvation leaves of early-senescing cultivars were characterized by inactivation of biologically active CKs, whereas in stay-green cultivars synthesis, activation, binding of and response to biologically active CKs were favoured. These results suggest that the homeostasis of biologically active CKs was the predominant leaf-inherent factor for cultivar differences in N starvation-induced leaf senescence and thus N efficiency.

## Highlight

The homeostasis of biologically active cytokinins was the predominant leaf-inherent factor for winter oilseed rape cultivar-differences in nitrogen starvation-induced leaf senescence and thus nitrogen efficiency.

## Introduction

Nitrogen efficiency of winter oilseed rape line-cultivars, defined as high grain yield under N limitation, has been primarily attributed to maintained N uptake during reproductive growth (N uptake efficiency) (Kamh *et al.*, 2005; Schulte auf’m Erley *et al.*, 2007, 2011; Berry *et al.* 2010). A characteristic of N-efficient cultivars is a functional stay-green phenotype during reproductive growth, expressed through delayed senescence of the older leaves accompanied with maintenance of photosynthetic capacity (Schulte auf’m Erley *et al.*, 2007). In many crop species, stay-green phenotypes are superior in yield formation particularly under abiotic stress conditions when senescence is prematurely induced, for instance by drought or N limitation (Gregersen *et al.*, 2013). Functional stay-green results from a delayed onset and/or progression of senescence (Thomas and Howarth, 2000). Thus, in contrast to cosmetic stay-green without any photosynthetic activity (Thomas and Howarth, 2000), functional stay-green requires more than the retention of green colour based on delayed or completely inhibited chlorophyll catabolism (Kusaba *et al.*, 2013).

Functional stay-green is an inheritable, mostly polygene-regulated quantitative trait (Wang *et al.*, 2012). But the underlying mechanisms are currently poorly understood. The onset of leaf senescence is accompanied by transcriptional regulation of many genes (Smart, 1994). Master-regulators of the gene-expression network during senescence are senescence-inducible transcription factors (TFs), which positively or negatively act on leaf senescence (Balazadeh *et al.*, 2008; Parlitz *et al.*, 2011). Many of these TFs directly or indirectly control the activation or repression of down-stream senescence-associated genes (SAGs) to fine-tune both the onset and rate of leaf senescence (Kusaba *et al.*, 2013). A hallmark of leaf senescence is the termination of photoassimilation. For functional stay-green, the maintenance of functional chloroplasts and thus photoassimilation is a prerequisite, a process that is under the control of nuclear as well as chloroplast encoded genes (Sakuraba *et al.*, 2012a, b; Rauf *et al.*, 2013).

Additional key regulators of leaf senescence are phytohormones (Lim *et al.*, 2007). Alterations in phytohormone, particularly cytokinin (CK), metabolism and signalling lead to functional stay-green phenotypes (Thomas and Ougham, 2014). Cytokinins are the most potent general antagonist of senescence (Wingler and Roitsch, 2008; Zwack and Rashotte, 2013). Although currently nothing is known about the underlying mechanisms for genotypic variation in functional stay-green under N starvation in winter oilseed rape, leaf-inherent and/or root-mediated CKs might be important factors. For functional stay-green of mature leaves in cotton plants root-derived CKs are important (Dong *et al.*, 2008; Dai and Dong, 2011), while leaf-inherent CK factors lead to functional stay-green in other plant species, exemplified by modification of leaf-inherent CK homeostasis in mature tobacco leaves (Gan and Amasino, 1995; del Mar Rubio-Wilhelmi *et al.*, 2014) as well as CK perception and signalling in mature *Arabidopsis thaliana* leaves (Kim *et al.*, 2006) or downstream targets in tomato (Albacete *et al.*, 2014) and tobacco leaves (Balibrea *et al.*, 2004).

An important aspect in addressing CK function is that CKs can be divided into biologically active CKs and biologically inactive CKs. The biologically inactive CKs are conjugated forms of the biologically active CKs and are used for storage and transport. In addition, the conjugation into biologically inactive CKs allows a fine-tuned regulation of the appropriate biologically active CKs levels. A subset of these conjugation reactions is reversible, enabling a rapid release of biologically active CKs from biologically inactive CK storage pools (Schmülling, 2004; Sakakibara, 2006).

To elucidate whether cultivar differences in N starvation-induced leaf senescence in winter oilseed rape are due to leaf-inherent factors and/or governed by root-mediated signals, a reciprocal grafting approach was applied in the present study using two cultivar pairs differing in N efficiency and N starvation-induced leaf senescence. To clarify the role of phytohormones for genotypic variation in functional stay-green, phytohormone levels were determined in roots, xylem sap and individual leaves in a complementary time-course experiment comprising the same four cultivars used for the grafting approach. In addition, the expression of selected genes involved in CK homeostasis, perception and signalling were analysed in the leaf tissue.

## Material and Methods

### Grafting experiment

#### Plant material

Based on a previous experiment comparing N starvation and leaf detaching as inducers of leaf senescence in 10 line-cultivars (cvs.), two cultivar pairs were selected for the reciprocal grafting approach. The cvs. NPZ-1 & NPZ-2 are breeding lines with a similar genetic background and reacted differently to N starvation and detaching, indicating root-derived factors. The commercial cvs. Apex and Capitol were selected since they did not differ in leaf senescence irrespective of the senescence inducer, suggesting leaf-inherent factors (Supplementary Fig. S1). Plants of both cultivar pairs were reciprocally-grafted or not-grafted and self-grafted as controls.

#### Grafting procedure

The seeds were germinated and the seedlings were grafted in a climate chamber (day/night 16/8h; temperature day/night 22/20°C; PAR 350 µmol m^-2^ s^-1^). The seeds were sown in substrate consisting of white peat, sand and perlite in the ratio of 3:1:2 (w:w:w). The substrate was limed to pH 6 using 4g l^-1^ limestone (85% calcium carbonate, Otterbeinkalk) and macro- and micronutrients were added using 0.5g l^-1^ Flory 3 (Euflor, Schrembeck, Germany) and 0.1g l^-1^ Flory 10 (Euflor, Schrembeck, Germany). Seven days after germination the hypocotyl reached a diameter of 1mm and the plants were grafted following a modified procedure described by Moroni (1997). The hypocotyls were cut horizontally, and roots and shoots fixed to each other via a well-fitting PVC-tube (PVC-Standard diameters: 1.020; 1.143; 1.295; 1.422mm, Spetec, Erding, Germany). The grafted plants were kept for 5 d under a wet tent. The humidification was gradually reduced to the ambient conditions in the climate chamber in daily steps. Afterwards the plants were acclimatized in the climate chamber for one day before they were transferred to hydroponics.

#### Hydroponic growing conditions

The plants were cultured in a greenhouse [assimilation light (16 klm) 16h; heating/ventilation temperature 22/20°C; shading at 15 klx solar radiation; relative humidity 80%] from 24 February to 4 April 2012. The roots of the plants were washed out of the substrate using deionized water. Two plants were transferred to a 6 l plastic pot and pre-cultured for 28 d at optimal N supply (2.0mM). The roots of the plants were separated by hand daily, to avoid intermingling of the root systems. The roots remained submerged during the separation to minimize root damage. The composition of the nutrient solution was 500 µM K_2_S0_4_, 250 µM KH_2_PO_4_, 325 µM MgSO_4_, 50 µM NaCl, 8 µM H_3_BO_3_, 0.4 µM MnSO_4_, 0.4 µM ZnSO_4_, 0.4 µM CuSO_4_, 0.1 µM Na_2_MoO_4_ and 40 µM Fe-EDDHA. During pre-culture Ca(NO_3_)_2_ and (NH_4_)_2_SO_4_ were used as N sources in the ratio of 9:1, and 10 µM C_2_H_4_N_4_ (dicyandiamide) was added to prevent nitrification. Prior to treatment one plant per pot was discarded, which allowed selection for homogeneity of the experimental plants. For N starvation the plants were grown at 0.1mM N [Ca(NO_3_)_2_] and 1.0mM CaSO_4_ to allow for optimum Ca nutrition. For optimal N nutrition the plants were cultured at 4.0mM N doubling the concentration used for pre-culture. After the start of treatment the nutrient solution was changed every second day. The experiment was completely randomized with four biological replications.

#### Non-destructive measurements, plant harvest and analysis

During the treatment the senescence status of the third and the fourth leaf, counted from the bottom to the top of the plant, was measured using non-destructive methods. The fifth leaf from the bottom of the plant was the youngest fully expanded leaf at the end of pre-culture. The chlorophyll content of the third and the fourth leaf was assessed by a portable chlorophyll meter (SPAD-502, Konica Minolta, Tokyo, Japan). Photosynthesis rate of the fourth leaf was measured using a portable gas exchange system (LI-6400, LI-COR, Lincoln, USA) at a photon flux density of 1000 µmol m^-2^ s^-1^ and an incoming CO_2_ concentration of 400 µmol m^-2^ s^-1^ to assess functional stay-green. The plants were harvested 12 d after start of treatment (DAT) and were separated into shoot and root, while the third and the fourth leaf were harvested separately. The leaf area was measured by a portable leaf area meter (LI-3100, LI-COR, Lincoln, USA). The leaf was divided along the midrib with a razor blade. One half was immediately frozen in liquid N_2_ and the other half was dried at 70°C until constant weight for dry weight determination. N concentrations of the dried and ground root, shoot and leaf material were determined using an elemental analyser (Vario EL, Elementar Analysensysteme, Hanau, Germany). For RNA extraction, the frozen material was ground using a mixer mill (MM 400, Retsch, Haan, Germany).

### Kinetics of N starvation-induced leaf senescence

#### Plant material and growing conditions

The kinetics of the development of N starvation-induced leaf senescence was investigated for the same four cvs.: NPZ-1, NPZ-2, Apex and Capitol. The seeds were germinated in the climate chamber as described above using a ‘sandwich’ method arranging the seeds between filter paper sandwiched between sponges and PVC-plates on both sides. The ‘sandwiches’ were placed into a box containing tap water. Seven days after germination the seedlings were cultured from 23 September to 3 November 2010 in the greenhouse in hydroponics as described above. The experiment was completely randomized with 3–4 biological replications.

#### Plant harvest and analysis

Plants were harvested after 0, 3, 5, 7, 10 and 12 d of N starvation and after 7 and 12 DAT of optimal N nutrition and divided into root, shoot and three previously marked individual leaves, which were the youngest fully expanded leaf at the end of pre-culture and the two older leaves. In most cases, the youngest fully expanded leaf at the end of pre-culture was the fifth leaf from the bottom of the plant. But sometimes it was the fourth leaf due to differences in development between the cultivars. During N starvation, the senescence status of the three leaves was measured using non-destructive methods as describe above, while photosynthesis rate was measured at a photon flux density of 400 µmol m^-2^ s^-1^. The harvest procedure, the sample preparation and the N analysis were performed as described above.

#### Xylem sap collection and analysis

Xylem exudates were collected at each harvest. The nutrient solution was changed 1h before starting xylem sap collection according to the N variants and additionally KCl (2.0mM) was added to enhance exudation. The plants were horizontally cut using a razor blade at the root collar and a well-fitting silicone tube was attached. After 5min the exudates were discarded and then collected for 1h. The collected exudates were kept on ice, protected from light and after determination of the exudate volume for the calculation of the metabolite xylem transport-rate, the exudates were stored at −20°C. For analysis the exudates were gently defrosted on ice and protected from light.

#### Phytohormone analysis

The levels of abscisic acid (ABA), jasmonic acid (JA), salicylic acid (SA) and of the cytokinins (CKs) *trans*-Zeatin (tZ), *cis*-Zeatin (cZ), dihydrozeatin (DHZ), isopentenyladenin (iP), *trans*-Zeatin*-O*-glucoside (tZOG) and *trans*-Zeatin*-O*-glucoside-riboside (tZOGR) were analysed in the root, the xylem sap and two individual leaves per plant (the second and third oldest harvested mature leaves at the end of pre-culture). Extractions were performed using 250mg of frozen and ground leaf and root tissue, or 100 µl xylem sap, after adding 4 μl of internal standard mix composed of deuterium labelled hormones. Phytohormone detection was performed on a UHPLC–MS/MS system consisting of a Thermo ACCELA pump (Thermo Scientific, Waltham/USA) coupled to a tempered HTC-PAL autosampler (CTC Analytics, Zwingen/Switzerland), and connected to a Thermo TSQ Quantum Access Max Mass Spectrometer (Thermo Scientific) with a heated electrospray ionization (HESI) interface, using a Nucleoshell-PFP column (2.7 μm, 100×2mm; Macherey-Nagel) according to Großkinsky *et al*. (2014).

### RNA isolation and cDNA synthesis

RNA was extracted from 100mg of frozen and ground leaf material with TRIsure™ (Bioline, London, UK) reagent according to the instructions of the manufacturer. RNA integrity was tested on 1% agarose gel electrophoresis and photometrically quantified (NanoPhotometer™, Implen, München, Germany). Two micrograms of RNA were applied to synthesize cDNA with the RevertAid™ H Minus First Strand kit (Fermentas, Waltham, USA). For the reaction the supplied random hexamer primer were used to synthesize the first strand cDNA of the *m*RNA according to the instructions of the manufacturer. Quality and quantity of cDNA was determined as for RNA.

### Primer design and qRT-PCR

The relative expression of the gene *SAG12-1* encoding a senescence-specific cysteine protease and selected genes involved in CK homeostasis and biological impact were analysed using qRT-PCR. Among these were genes related to synthesis (isopentenyltransferase—*IPT*), to the reversible and irreversible glycosylation (uridine diphosphate glycosyltransferase—*UGT*) converting biologically active CKs to biologically inactive but activatable CKs or biologically inactive CK forms, respectively, to the release of highly biologically active CK nucleobases from their ribosides with lower biological activity (cytokinin riboside 5′-monophosphate phosphoribohydrolase—*LOG*), to the breakdown of biologically active CKs (cytokinin oxidase/dehydrogenase—*CKX*), and to the perception of and response to biologically active CKs (histidine kinase 3—A*HK3*; response regulator 2—A*RR2*). The gene *EF1-alpha* encoding an elongation factor was used as reference gene (Desclos *et al.*, 2008). The initial selection of the analysed genes related to cytokinin homeostasis was based on the availability of sequence information in *A. thaliana* and *B. napus*. The final selection of the respective isogenes was based on the detectability of the respective gene expression in leaf tissues. A gene was detected when the fluorescence signal of its amplicon exceeded the threshold within 50 PCR cycles. For the design of the primer pairs *B. napus* sequences were used. If no suitable primer pair could be designed on the *B. napus* sequence the primer pair was designed on the sequence of the *A. thaliana* homologue gene. The *B. napus* sequences were retrieved from the NCBI public database and The Gene Index Project at the Dana-Farber Cancer Institute (DFCI; http://compbio.dfci.harvard.edu/tgi/plant.html). The *A. thaliana* sequences were obtained from the Arabidopsis Information Resource Version 10 (TAIR; http://www.arabidopsis.org). Primer pairs were designed using PrimerQuest (Integrated DNA Technologies, Coralville, USA) for genes having NCBI accession numbers, or using QuantPrime (Arvidsson *et al.*, 2008) for genes having DFCI or TAIR accession numbers (Supplementary Table S1). PCR reactions for qRT-PCR were performed as described by Koeslin-Findeklee *et al.* (2015). The mean relative expression and the standard error of the qRT-PCR results were calculated based on the 2^-ΔΔCt^-method by Livak and Schmittgen (2001).

### Statistical analysis

The statistical analysis was performed using the statistic software SAS version 9.2 (SAS Institute, Cary, USA). For the comparison of the two grafting experiments the data were ranked using the PROC RANK procedure followed by an analysis of variance (ANOVA) using the PROC GLM procedure. For analysis of data from the grafting experiment and the experiment investigating the kinetics of the development of N starvation-induced leaf senescence the ANOVA was calculated using the PROC GLM procedure. For the ANOVA the Type III sum of squares was applied, if an unequal number of replicates occurred. Multiple comparisons of means were calculated by the MEANS statement of the PROC GLM procedure. For all tests of significance a *P*-value of 0.05 was used and the *P*-values were Bonferroni-Holm adjusted. Curves were fitted using the graphic software SIGMA PLOT version 11 (Systat software, San Jose, USA). The relative qRT-PCR data were analysed using the %QPCR MIXED macro after Steibel *et al.* (2009) based on the PROC MIXED procedure.

## Results

### Grafting experiment

To investigate whether cultivar differences in N starvation-induced leaf senescence are due to leaf-inherent factors and/or governed by root-mediated signals, a reciprocal grafting experiment was performed using two cultivar pairs differing in N efficiency and N starvation-induced senescence described under *Plant material* above. The experiment was performed twice. Since these experiments did not differ in N starvation-induced leaf senescence (Supplementary Table S2), the results of only one experiment are shown.

#### Chlorophyll content and photosynthesis rate in senescing leaves

**Fig. 1.**
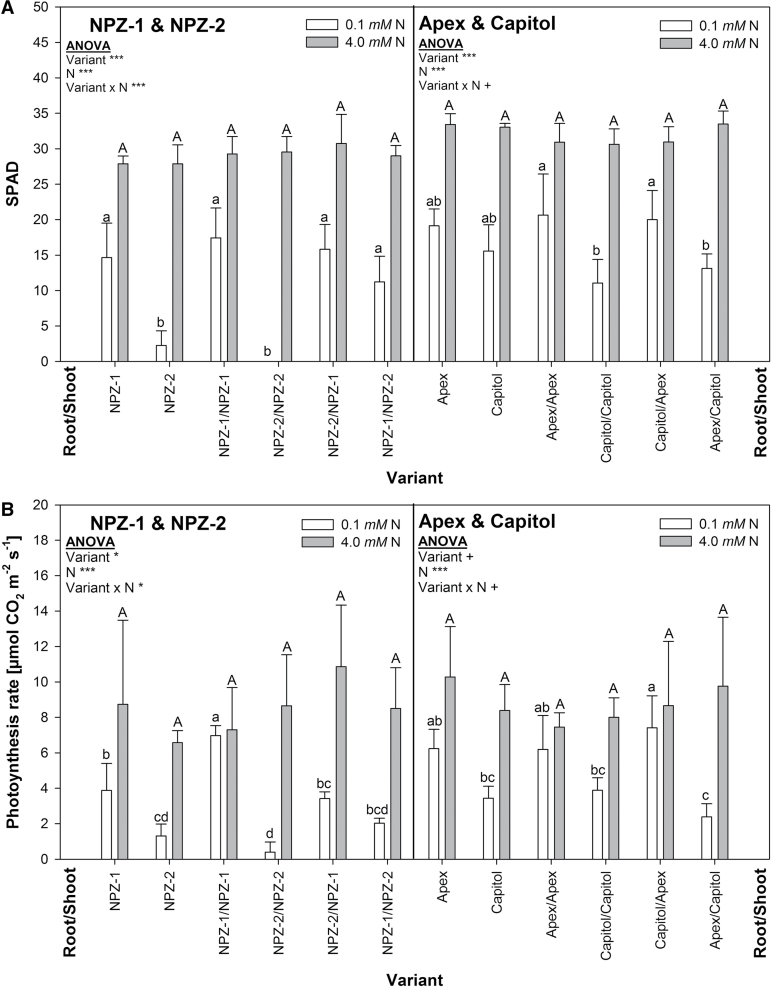
(A) SPAD values and (B) photosynthesis rate of leaf 4 in non-grafted, self-grafted and reciprocally-grafted plants of the winter oilseed rape cultivars NPZ-1 and NPZ-2 (left), and Apex and Capitol (right), grown in hydroponics after 12 d of N starvation (0.1mM N) or optimal N supply (4.0mM N). The plants were pre-cultured for 28 d at 2.0mM N. Different letters on top of the columns indicate differences between the variants (*P*<0.05). ANOVA: +, *, *** indicate significant differences at *P*<0.10, *P*<0.05, *P*<0.001, respectively. The error bars represent the standard deviations of the means (*n*=3**–**4).

Since for both cultivar pairs the overall statistical analysis of cholorophyll content (represented by SPAD values) revealed that for all variants the fourth leaf (‘leaf 4’) reacted to N starvation, although delayed (significant leaf effect), in exactly the same way as the third leaf (no significant variant × leaf × N interaction, Supplementary Table S3), only the results for leaf 4 are presented. At optimal N supply the SPAD values of all variants were high and did not differ (Fig. 1A). The SPAD values declined after 12 days of N starvation, reflecting senescence induction, and highly significant differences existed between the variants. The non-grafted controls and the self-grafted plants showed the expected differences in senescence within the cultivar pairs: cvs. Apex and NPZ-1 remained greener (higher SPAD values) than the cvs. Capitol and NPZ-2, although the controls of cvs. Apex and Capitol differed only in tendency. The leaf SPAD value depended primarily on the origin of the shoot for the reciprocal grafts of cvs. Apex and Capitol, and cvs. NPZ-1 and NPZ-2: the cv. Apex shoot (significantly) and the cv. NPZ-1 shoot (only in tendency), conferred delayed leaf senescence when grafted on the cv. Capitol and cv. NPZ-2 roots, respectively. The lack of significance was due to an unusually high SPAD value of the cv. NPZ-2 shoot on the cv. NPZ-1 root in this experiment which might be interpreted as a positive effect of the cv. NPZ-1 root. However, this result could not be reproduced in the second repetition of the experiment (not shown). As an additional indicator of the leaf senescence status photosynthesis was measured (Fig. 1B). Photosynthesis reacted more sensitively to N starvation than SPAD with a significant decline. No differences existed between the variants at high N supply. Under N starvation leaf photosynthesis of non-grafted and self-grafted plants of cv. Capitol (in tendency) and cv. NPZ-2 (significantly) was lower than of cv. Apex and cv. NPZ-1, respectively. As for SPAD, photosynthesis of the grafted plants was determined mainly by the origin of the shoot: the cv. Apex shoot conferred less decline of photosynthesis than the cv. Capitol shoot when reciprocally grafted on the respective roots. For the cultivar pair NPZ-1 and NPZ-2 a comparative shoot effect was visible only in tendency for the reasons mentioned above for SPAD.

#### Expression of senescence-specific cysteine protease gene SAG12-1 in senescing leaves

**Fig. 2.**
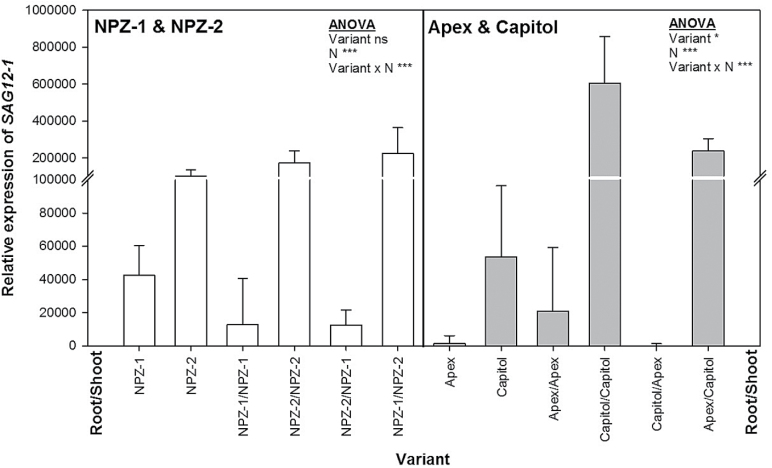
Relative expression (2^-ΔΔCt^) of *SAG12-1* in leaf 4 in non-grafted, self-grafted and reciprocal-grafted plants of the winter oilseed rape cultivars NPZ-1 and NPZ-2 (left), and Apex and Capitol (right), grown in hydroponics after 12 d of N starvation (0.1mM N). The plants were pre-cultured for 28 d at 2.0mM N. The data are shown relative to the control (4.0mM N) harvested at the same day. ANOVA: *** indicate significant differences at *P*<0.001; ns, non-significant. The error bars represent the standard errors of the means (*n*=3**–**4).

As a further senescence marker, the relative expression of *SAG12-1* coding for a senescence-specific cysteine protease was determined. *SAG12-1* expression proved to be a much more sensitive indicator than SPAD and photosynthesis (compare Figs 1 and 2). The lower upregulation by N starvation of *SAG12-1* in the non-grafted and self-grafted cvs. Apex and NPZ-1 confirmed a delayed leaf senescence as compared to cvs. Capitol and NPZ-2. The dominant role of the shoot origin in N starvation-induced leaf senescence was well reflected by *SAG12-1* expression in leaves of the grafted plants, which was only significantly increased when cv. Capitol and cv. NPZ-2 were used as shoots.

#### Specific leaf N content in senescing leaves

**Fig. 3.**
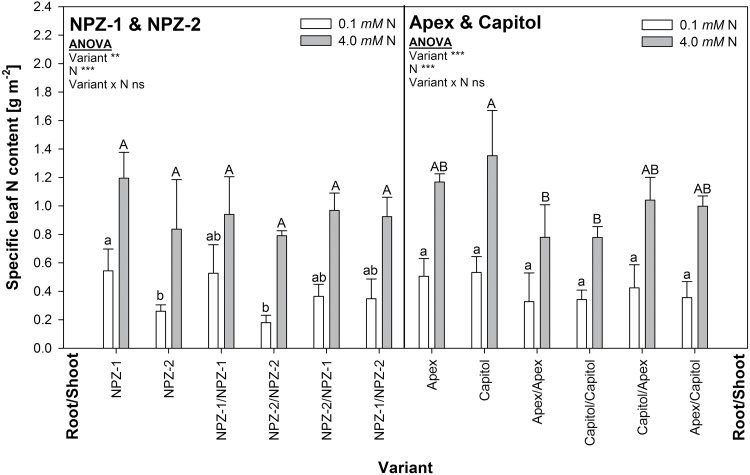
Specific leaf N content of leaf 4 in non-grafted, self-grafted and reciprocally-grafted plants of the winter oilseed rape cultivars NPZ-1 and NPZ-2 (left), and Apex and Capitol (right), grown in hydroponics after 12 d of N starvation (0.1mM N) or optimal N supply (4.0mM N). The plants were pre-cultured for 28 days at 2.0mM N. Different letters on top of the columns indicate differences between the variants (*P*<0.05). ANOVA: **, *** indicate significant differences at *P*<0.01 and *P*<0.001, respectively; ns, non-significant. The error bars represent the standard deviations of the means (*n*=3–4).

The cultivar differences in N starvation-induced leaf senescence could be due to differences in the depletion of the leaf N content. The specific leaf N content of leaf 4 significantly declined after 12 days of N starvation as compared to continuous high N supply for all cultivars and variants (Fig. 3). The non-grafted (significantly) and the self-grafted (tendentially) stay-green cv. NPZ-1 showed a higher specific leaf N content under N starvation as compared to the early-senescing counterpart cv. NPZ-2. However, no significant differences existed between the cvs. Apex and Capitol.

Under maintained high N supply, the cvs. NPZ-1 and NPZ-2 did not differ in the specific leaf N contents between the variants. In contrast, for the cultivar pair Apex and Capitol the specific leaf N content of the non-grafted was generally higher than that of the grafted plants. This difference was even significant between the non- and self-grafted plants of cv. Capitol.

### Development of N starvation-induced leaf senescence

To further clarify whether root-to-shoot communication and which leaf-inherent factors are decisive for the cultivar differences in stay-green, phytohormone levels were analysed in roots, xylem sap and individual leaves in a complementary time-course experiment with the same four cultivars used for the grafting approach. In this experiment, the cultivars also showed the established cultivar-specific responses to N starvation characterizing the cvs. NPZ-1 and Apex as stay-green and the cvs. NPZ-2 and Capitol as early-senescing (Koeslin-Findeklee *et al.*, 2015).

#### Plant phytohormone status

Phytohormones play a major role in root-to-shoot communication and in the control of leaf senescence. Therefore, the levels for the phytohormones SA, JA, ABA and CKs were analysed in roots, xylem exudates and leaves as affected by N starvation and cultivar. Only the results of the second oldest harvested leaf are presented since the statistical analysis did not reveal a consistent difference between the two analysed individual leaves (Supplementary Tables S4, S5). To identify a possible relationship between the root pool and the specific content of mature leaves showing leaf senescence during the treatment period, the root content, the xylem transport rate and the specific leaf contents were determined. Despite significant differences for some phytohormones between cultivars (Supplementary Tables S4, S5), only the measured biologically inactive but activatable CKs (iCKs) tZOG and tZOGR responded in a consistent way in relation to the cultivar-specific differences in N starvation-induced leaf senescence. Therefore, the following results showing the effects of treatment duration and N supply are means over the four cultivars.

The root SA content increased under both N supplies, reflecting the gain and differences in root biomass during the N treatment period (Supplementary Fig. S2A). The SA xylem transport rates did not differ either between the N supplies or the treatment times. The leaf specific SA content increased during the treatment time from 7 until 12 DAT independent of the N supply (no significant N effect). Also the JA root content increased until 12 DAT under both N supplies (Supplementary Fig. S2B). In contrast to SA the JA root content was higher under N starvation than with sufficient N supply at 12 DAT. The JA xylem transport rate did not show significant N supply and treatment-time effects. Up to 7 DAT the specific JA leaf content was neither affected by treatment duration nor by N supply. But after 7 DAT the JA content significantly increased under N starvation while at sufficient N supply it decreased (significant N × DAT interaction). The ABA root contents apparently increased more with treatment duration under high N supply than under N starvation, but this difference was not significant (Supplementary Fig. S2C). The ABA transport rate in the xylem increased significantly during the time-course of the treatment under sufficient N supply, but not under N starvation. The specific ABA content in the leaf tissue increased until 7 DAT for both N supplies. At 12 DAT, the ABA content was significantly higher in N-starved leaves, because it remained at high level, whereas it deceased under sufficient N.

Among the phytohormones, CKs play a major role in both root-to-shoot communication and leaf senescence. The individually measured CKs were grouped according to their biological activity (Schmitz *et al.*, 1972; Schmülling, 2004) into biologically active (aCKs) and biologically inactive but activatable CKs (iCKs). The aCKs (tZ, iP, DHZ, cZ, tZR) are of particular importance in relation to stay-green. The overall statistical analysis (Supplementary Table S5) revealed that in spite of significant differences between the individual aCKs neither the cultivar nor the N supply affected the aCKs. Moreover, no systematic pattern was apparent in relation to cultivar, treatment duration (DAT) and N supply (no significant cultivar × DAT × N × CKs interaction). Therefore, the individual aCK data were pooled for the root, xylem sap and leaf tissue.

**Fig. 4.**
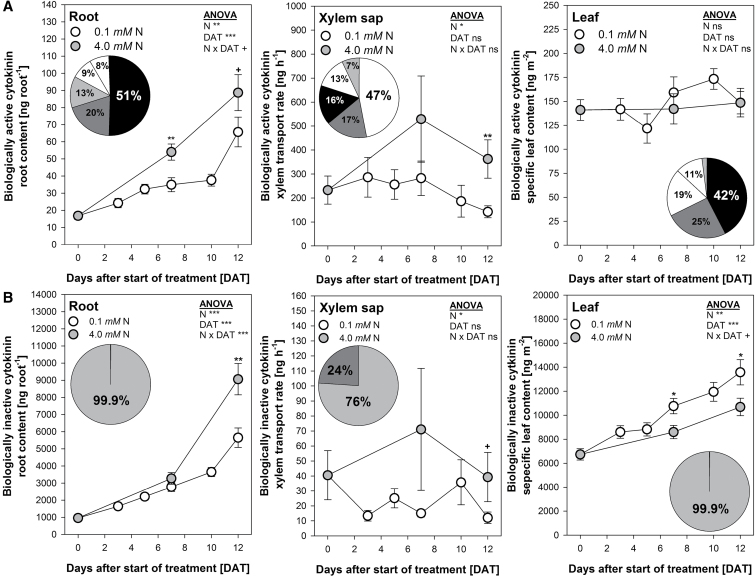
(A) Biologically active cytokinins (tZ, iP, DHZ, cZ, tZR) and (B) biologically inactive but activatable cytokinins (tZOR, tZORG) in the root, the xylem sap and the second oldest harvested mature leaf of the winter oilseed rape cultivars NPZ-1, NPZ-2, Apex and Capitol grown in hydroponics during 12 d N starvation (0.1mM) or optimal N supply (4.0mM). The plants were pre-cultured for 28 d at 2.0mM N. Pie chart in A: tZ (black), DHZ (dark grey), tZR (grey), cZ (white), iP (hatched). Pie chart in B: tZOG (grey), tZROG (dark grey). ANOVA: +, *, **, *** indicate significant differences at *P*<0.10, *P*<0.05, *P*<0.01, *P*<0.001, respectively; ns, non-significant. At 7 and 12 DAT: +, *, ** indicate differences between the N supplies at *P*<0.10, *P*<0.05, *P*<0.01, respectively. The error bars (visible only when greater than the symbols) represent the standard errors of the means across the four cultivars (*n*=3–4).

In the root and leaf tissue, tZ was the most abundant aCK (51% and 42%, respectively; Fig. 4A). However, in the xylem sap iP was the most abundant aCK (47%). In the root the aCK content increased until 12 DAT under both N supplies, but significantly more under sufficient N supply primarily due to increasing root biomass. The xylem transport-rate of aCKs remained stable over the treatment time (no significant DAT effect), but was significantly enhanced under high N supply. The specific leaf content of the aCKs was affected neither by the duration of N starvation (DAT) nor by N supply and remained at a constant level.

The glycosylation of biologically active CKs (aCKs) to *O*-glycosides (iCKs) leads to their reversible inactivation. In the leaf tissue this inactivation of the aCKs might be of importance for cultivar differences in N starvation-induced leaf senescence. The iCKs constitute the majority of the total CKs in root and leaf tissues (Fig. 4B). Among the iCKs, tZOG represented more than 99% of that fraction in root and leaf tissues independent of N supply and duration of N starvation (DAT, not shown). In the xylem, iCKs were also mostly transported as tZOG (76%). The highly significant influence of the duration of N starvation (DAT) on the root content of iCKs was due to root growth during the treatment period rather than root concentrations (not shown). The xylem transport rate of iCKs increased during the treatment period only at high N supply. The lack of a significant DAT effect (ANOVA) may be explained by the high variation of the mean at 7 DAT at high N supply. In the leaf tissue, the iCKs significantly increased during the treatment period. The increase was steeper under N starvation than at optimum N supply (significant N × DAT interaction).

**Fig. 5.**
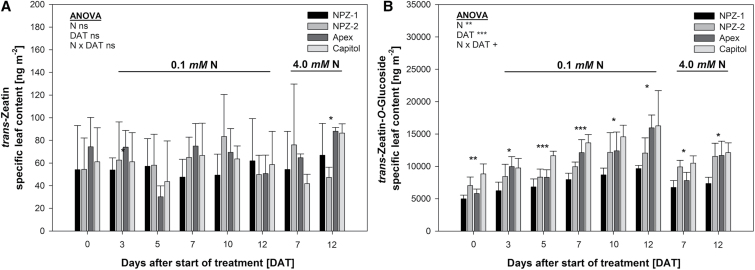
(A) *trans*-Zeatin and (B) *trans*-Zeatin-*O*-Glucoside specific leaf content of the second oldest harvested mature leaf of the winter oilseed rape cultivars NPZ-1, NPZ-2, Apex and Capitol grown in hydroponics during 12 d N starvation (0.1mM) or optimal N supply (4.0mM). The plants were pre-cultured for 28 d at 2.0mM N. ANOVA: +, *, **, *** indicate significant differences at *P*<0.10, *P*<0.05, *P*<0.01, <0.001, respectively; ns, non-significant. *, **, *** above the columns indicate significant differences between the cultivars at *P*<0.05, *P*<0.01, *P*<0.001, respectively. The error bars represent the standard deviations of the means (*n*=3–4).

The overall statistical evaluation revealed that the iCKs but not the aCKs showed a consistent difference between the cultivars and N supplies in the leaf tissue (significant cultivar × DAT × N interaction, Supplementary Table S5), evident by the cultivar-specific specific leaf contents of the dominating individual aCKs (tZ) and iCKs (tZOG) (Fig. 5). The specific leaf content of tZ was not affected by N supply and the duration of N starvation (ANOVA). Differences between cultivars were only significant in high N leaves at 12 DAT. In contrast, the tZOG specific leaf content highly significantly increased during treatment duration to a larger extent in N-starved than in N-sufficient leaves (significant N × DAT interaction). The early-senescing cvs. NPZ-2 and Capitol mostly showed higher tZOG contents than their stay-green counterpart cvs. NPZ-1 and Apex, both under N starvation and at high N supply.

#### Regulation of cytokinin homeostasis-related genes in senescing leaves

To investigate the importance of *de novo* synthesis, metabolic inter-conversion and breakdown of biologically active CKs for cultivar differences in functional stay-green during N starvation, the regulation of genes assigned to these processes were analysed.

**Fig. 6.**
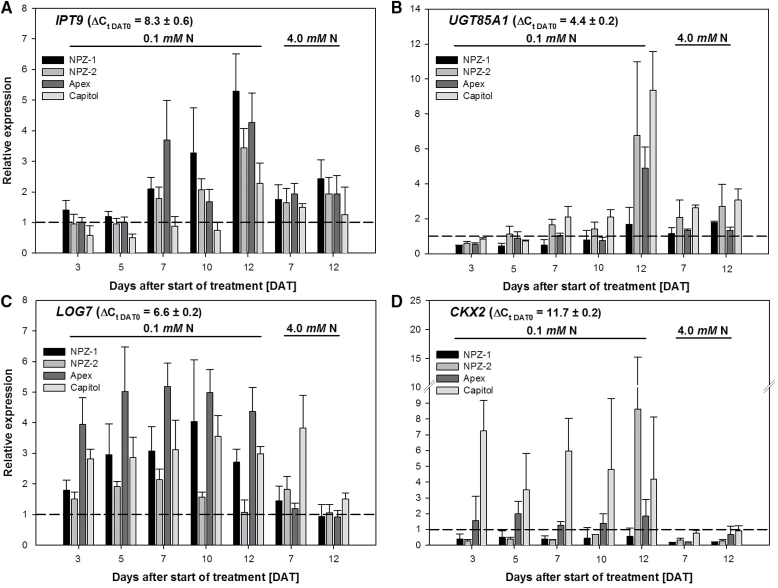
Relative expression (2^-ΔΔCt^) of (A) the isopentenyltransferase (IPT) gene *IPT9*, (B) the uridine diphosphate glycosyltransferases (UGT) gene *UGT85A1*, (C) the cytokinin ribosid 5′ monophosphate phosphoribohydrolase gene *LOG7* and (D) the cytokinin oxidase (CKX) gene *CKX2*, in the second oldest harvested mature leaf of the winter oilseed rape cultivars NPZ-1, NPZ-2, Apex and Capitol grown in hydroponics during 12 d N starvation (0.1mM) or optimal N supply (4.0mM). The plants were pre-cultured for 28 days at 2.0mM N. The data are shown relative to the cultivar-specific control at DAT 0 (dashed line). The error bars represent the standard errors of the means (*n*=3–4). ± indicates the standard deviation of the mean for the ΔC_t_ across the four cultivars.

The first step of CK synthesis is catalysed by isopentenyltransferases (IPTs). Transcripts of the three *IPT* genes *IPT2, IPT5* and *IPT9* were detected in the leaf tissue under both N supplies. The cultivars generally showed a similar basal expression (DAT 0), but the basal expression levels (ΔC_t_) differed greatly between the three IPT genes, in ascending order: *IPT2* < *IPT9* < *IPT5* (Supplementary Fig. S3). Among the three genes *IPT9* was most clearly upregulated (up to 5-fold) under N starvation after 7 until 12 DAT and at most of the harvest days, at least in tendency, in a cultivar-specific way (Fig. 6A). *IPT9* showed higher upregulation in the stay-green cvs. NPZ-1 and Apex as compared to their respective early-senescing counterpart cvs. NPZ-2 and Capitol. N starvation also generally enhanced *IPT2* upregulation and at most of the harvest days, at least in tendency, a cultivar-specific higher upregulation occurred in the stay-green cultivars as compared to their respective early-senescing counterparts (Supplementary Fig. S3).

The *O*-glycosylation of biologically active CKs leading to their reversible inactivation is catalysed by the uridine diphosphate glycosyltransferases (UGTs). In the leaf tissue under both N supplies transcripts were detected of the *UGT* genes *UGT73C1, UGT73C5* and *UGT85A1*, which catalyse, and *UGT73C4,* which possibly catalyses, the formation of CK *O*-glycosides. The cultivars generally showed a similar basal expression (DAT 0), but the basal expression level (ΔC_t_) differed greatly between the four UGT genes, in ascending order: *UGT73C1* < *UGT73C4* < *UGT73C5* < *UGT85A1* (Supplementary Fig. S4). *UGT85A1* with the highest basal expression level clearly responded to N starvation and showed higher upregulation in the early-senescing cvs. NPZ-2 and Capitol than in their respective stay-green counterpart cvs. NPZ-1 and Apex (Fig. 6B). The *UGT* genes *UGT73C4* and *UGT73C5* also responded to N starvation even more sensitively and showed a consistent cultivar-specific regulation pattern corresponding to the respective senescence phenotype (Supplementary Fig. S4B, C).

The release of the bioactive CK nucleobases from their CK riboside 5′ monophosphates is catalysed in a single step by the enzyme cytokinin riboside 5′-monophosphate phosphoribohydrolase, which is encoded by *LONELY GUY* (*LOG*). The cultivars generally showed a similar basal expression (DAT 0), but the basal expression level (ΔC_t_) differed greatly depending on the gene, in decreasing order LOG1 > LOG7 > LOG4 > LOG5 (Supplementary Fig. S5). Among the detected *LOG* genes *LOG7* showed a mostly consistent cultivar-specific response to N starvation across the treatment duration (Fig. 6C). *LOG7* showed higher levels of upregulation in the stay-green cvs. NPZ-1 and Apex as compared to their early-senescing counterpart cvs. NPZ-2 and Capitol (Fig. 6C). Considering the high levels of upregulation of *LOG7* in cv. Capitol after 7 days of high N treatment, the upregulation by N starvation is even less than suggested by the ΔΔC_t_ values shown in Fig. 6C, increasing the difference in comparison to cv. Apex. Also *LOG4* and *LOG5* clearly responded to N starvation (Supplementary Fig. S5). But only *LOG4* showed a consistent cultivar-specific regulation pattern comparable to *LOG7*.

The catabolism of biologically active CKs is tightly regulated by cytokinin oxidase/dehydrogenases (CKXs). The five analysed *CKX* genes generally showed a similar basal expression between the cultivars (DAT 0; Supplementary Fig. S6). But the basal expression level (ΔC_t_) differed depending on the gene in ascending order *CKX6* < *CKX7* < *CKX1* < *CKX*3 < *CKX*2. *CKX1*, *CKX2* and *CKX3* were generally upregulated by N starvation. Among all *CKX* genes, *CKX2* with the lowest basal expression level was most strongly expressed by N starvation in a highly cultivar-specific way (Fig. 6D). The early-senescing cv. Capitol showed the highest upregulation independent of the duration of N starvation, whereas stay-green cv. Apex only weakly responded to N starvation. *CKX2* was downregulated in the cvs. NPZ-1 and NPZ-2 until DAT 12 when the early-senescing cv. NPZ-2 strongly upregulated *CKX2* expression.

#### Regulation of cytokinin perception and signalling-related genes in senescing leaves

**Fig. 7.**
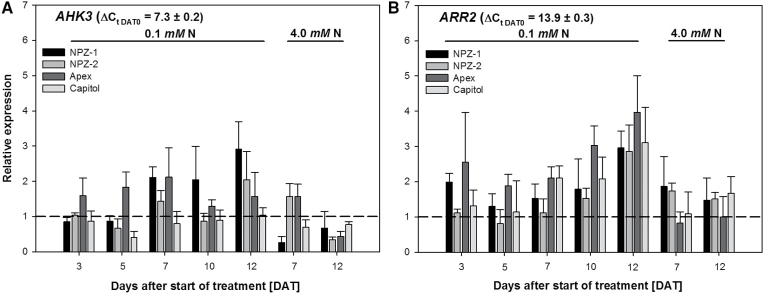
Relative expression (2^-ΔΔCt^) of (A) the histidine kinase gene A*HK3* and (B) the response regulator gene *ARR2* in the second oldest harvested mature leaf of the winter oilseed rape cultivars NPZ-1, NPZ-2, Apex and Capitol grown in hydroponics during 12 d N starvation (0.1mM) or optimal N supply (4.0mM). The plants were pre-cultured for 28 d at 2.0mM N. The data are shown relative to the cultivar-specific control at DAT 0 (dashed line). The error bars represent the standard errors of the means (*n*=3–4). ± indicates the standard deviation of the mean for the ΔC_t_ across the four cultivars.

For the action of CKs on plant processes the local perception of and response to biologically active CKs, the binding to specific receptors and the activation of response regulators are necessary. The CK receptor encoded by the histidine kinase receptor *AHK3* and the downstream response regulator encoded by *ARR2* are involved in the CK-regulated leaf longevity. Basal expression levels did not differ between the cultivars for both genes (DAT 0; Fig. 7). *AKH3* upregulation increased with the duration of N starvation in a cultivar-specific way (Fig. 7A). Considering the downregulation during the treatment period at high N supply, *AHK3* was most strongly upregulated by N starvation in the stay-green cv. NPZ-1, particularly in comparison to its early-senescing counterpart cv. NPZ-2. A comparable higher expression was observed in the stay-green cv. Apex as compared to cv. Capitol, which did not show any upregulation. *ARR2* upregulation generally increased with the duration of N starvation in all cultivars (Fig. 7B). As for *AHK3*, *ARR2* was on the whole consistently more upregulated under N starvation in the leaves of the stay-green cvs. NPZ-1 and Apex than in the leaves of cvs. NPZ-2 and Capitol.

## Discussion

Nitrogen efficiency of line cultivars has been primarily attributed to maintained N uptake during reproductive growth (N uptake efficiency) in combination with delayed senescence of the older leaves accompanied with maintained photosynthetic capacity (functional stay-green) (Schulte auf’m Erley *et al.*, 2007). But currently it is not clear whether higher root growth and N uptake during early reproductive growth of N-efficient cultivars are causally related, and when related, if delayed leaf senescence is the cause or the consequence of maintained root growth.

The experimental approaches were based on experimental evidence showing that the cvs. NPZ-1 and NPZ-2 reacted differently in leaf senescence to N starvation and leaf detaching, indicating root-derived factors. In contrast, the cvs. Apex and Capitol reacted in the same way to the senescence inducers, indicating leaf-inherent factors (Supplementary Fig. S1). In order to unequivocally clarify whether the cultivar differences in N starvation-induced leaf senescence are root-mediated and/or leaf-inherent a reciprocal grafting approach was applied.

The senescence status of older leaves of the reciprocal grafts after 12 days of N starvation, assessed by SPAD (Fig. 1A), photosynthesis (Fig. 1B) and, most prominently, by *SAG12-1* expression (Fig. 2) suggest that the cultivar differences between the stay-green cvs. NPZ-1 and Apex and their respective early-senescing counterpart cvs. NPZ-2 and Capitol were based primarily on leaf-inherent factors rather than on root-derived signals. *SAG12-1* has been used as a suitable molecular leaf-senescence marker in winter oilseed rape (Etienne *et al.*, 2007) and proved to be a highly sensitive marker for the detection of cultivar differences in N starvation-induced leaf senescence (Koeslin-Findeklee *et al.*, 2014, 2015). The encoded cysteine protease is involved in protein breakdown for N remobilization during senescence and is controlled on the transcription level (Grbić, 2003). No cultivar differences existed under N starvation at 12 DAT in shoot and root N uptake (Supplementary Fig. S7) as well as N concentrations (Supplementary Fig. S8). Therefore, it is unlikely that the genotypic variation in functional stay-green was based on differences in N uptake or N utilization efficiency.

Since N content and photosynthetic activity of leaves are closely related (Evans, 1989), the specific leaf N content might be an important leaf-inherent factor for genotypic variation in functional stay-green. Particularly under N starvation, maintained photosynthetic activity could be related to the remaining specific leaf N content (Schulte auf’m Erley *et al.*, 2007; Koeslin-Findeklee *et al.*, 2014). But in the present study, cultivar differences in N starvation-induced leaf senescence at early stages with declined but still substantial photosynthesis rates, were not reflected by the specific leaf N content (Fig. 3). Solely the specific leaf N contents of the severely senescent non- and self-grafted cv. NPZ-2 at 12 DAT were either significantly or by trend lower as compared to the respective variants of cv. NPZ-1. This may explain the generally significant positive correlation between the specific leaf N content and photosynthesis rate (r^2^ = 0.20**; Supplementary Fig. S9). But only 20% of the variation in photosynthesis rate could be attributed to the specific leaf N content. Consequently, the development of cultivar differences in functional stay-green under N starvation depended primarily on other leaf-inherent factors than the specific leaf N content. This is in agreement with the results of Schulte auf’m Erley *et al.* (2010) for cultivar differences in N starvation-induced leaf senescence in maize.

Phytohormones are master-regulators of the leaf senescence process, in which SA, JA and ABA promote senescence whereas CKs delay it (Lim *et al.*, 2007). Supporting the leaf-inherent control of N starvation-induced leaf senescence, the root contents of SA, JA, ABA, aCKs and iCKs and their transport rates in the xylem in the complementary time-course experiment did not suggest a role of root-derived phytohormone signals for cultivar differences in functional stay-green (Fig. 4A, B, Supplementary Fig. S2A–C). Under N starvation SA as well as JA accumulated in the leaf, but only at later stages of N starvation from 7 until 12 DAT (Supplementary Fig. S2A, B), suggesting that SA and JA were involved in the regulation of N starvation-induced leaf senescence only at advanced stages. Thus a major role of SA and JA as leaf-inherent factors for the development of genotypic variation in functional stay-green under N starvation is unlikely.

In the complementary time-course experiment leaf-photosynthesis rapidly declined after exposure to N starvation as shown previously (Koeslin-Findeklee *et al.*, 2015). Abscisic acid regulates the stomatal conductance, which influences the photosynthesis rate (He *et al.*, 2005). Under N starvation, ABA accumulation in the leaf was accompanied with a decrease of the photosynthesis rate (not shown). Thus, ABA accumulation in the leaf under N starvation might be an important leaf-inherent factor for the termination of photoassimilation and thus the final stage of the leaf senescence process. Nevertheless, the cultivar differences in functional stay-green could not be explained by differences in the specific ABA leaf content (Supplementary Table S4).

With regard to functional stay-green CKs are of particular importance (Balibrea et al., 2004, del Mar Rubi-Wilhelmi et al., 2014). In the present study, the measured aCKs remained at a constant level in the complementary time-course experiment, independent of N supply and cultivar (Fig. 4A). Only the iCKs responded in a N starvation and cultivar-specific way: the stay-green cvs. NPZ-1 and Apex showed a lower accumulation of iCKs in senescing leaf tissue as compared to their respective early-senescing counterpart cvs. NPZ-2 and Capitol (Fig. 4B). This suggests that CK homeostasis was a decisive leaf-inherent factor for genotypic variation in functional stay-green under N starvation. Hence the regulation of genes assigned to CK *de novo* synthesis, inter-conversion and breakdown were analysed in the same experiment. The rate-limiting step of CK biosynthesis is catalysed by the enzyme isopentenyltransferase (IPT) (Schmülling, 2004; Sakakibara, 2006). Among the detected *IPT* genes in the leaf, particularly the tRNA IPTs *IPT9* (Fig. 6A) and *IPT2* (Supplementary Fig. S3A) were upregulated by N starvation and in a cultivar-specific way. Both IPT isoforms catalyse the formation of cZ derived from tRNA degradation (Schmülling, 2004; Sakakibara, 2006). Although the cZ content in the leaf did not change, the *IPT* gene expression patterns indicated a higher leaf-inherent cZ biosynthesis in the stay-green cvs. NPZ-1 and Apex as compared to their respective early senescing counterpart cvs. NPZ-2 and Capitol under N starvation. Despite the fact, that cZ is less biologically active (Schmitz *et al.*, 1972) and its biosynthesis requires a high tRNA turnover (Sakakibara, 2006), it might have significance for genotypic variation in functional stay-green, because during senescence a high tRNA turnover occurs (Buchanan-Wollaston, 1997) and cZ is less susceptible to degradation (Sakakibara, 2006). Moreover, the results of Miyawaki *et al.* (2004) support the view that in senescing leaves tRNA may be an important source for the leaf-inherent synthesis of aCKs.

In addition to the biosynthesis of biologically active CKs the spatial and temporal fine-regulated metabolic inter-conversion of CKs decisively affects the impact of CKs on plant processes (Schmülling, 2004; Sakakibara, 2006). The glycosylation of biologically active CKs lead to their inactivation. This is mediated by specific CK uridine diphosphate (UDP) glycosyltransferases (UGTs) forming either reversible *O*-glycosides or irreversible *N*-glycosides (Schmülling, 2004; Sakakibara, 2006). The UGTs encoded by *UGT73C1, UGT73C5* and *UGT85A1* particularly catalyse the formation of tZOG and DHZOG, whereas *UGT76C1* is involved in the formation of irreversibly inactivated *N*-glycosides of tZ, DHZ, iP and cZ (Hou *et al.*, 2004; Wang *et al.*, 2013). Among the CK-specific UGTs, UGT85A1 has been identified as the key enzyme for the formation of tZOG in *A. thaliana*. The transcript amount correlated with the accumulation of tZOG in plant tissues (Hou *et al.*, 2004; Jin *et al.*, 2013). Transcripts of *UGT85A1* were most abundant among the detectable *UGT* genes in winter oilseed rape leaves (Supplementary Fig. S4). In the complementary time-course experiment the expression pattern of *UGT85A1* reflected the cultivar-specific tZOG accumulation in the leaf under both N supplies (Fig. 6B): *UGT85A1* was more highly expressed in the leaf tissue of cv. NPZ-1 as compared to cv. NPZ-2, as well as in that of cv. Apex as compared to cv. Capitol, under both N starvation and optimal N supply. In addition the cultivar-specific higher upregulation of *UGT73C4* and *UGT73C5*, particularly under N starvation, corresponded to the greater accumulation of iCKs in the leaf of the early-senescing cvs. NPZ-2 and Capitol as compared to their respective stay-green counterpart cvs. NPZ-1 and Apex (Supplementary Fig. S4B, C). In contrast, *UGT73C1* was generally downregulated (Supplementary Fig. S4A). Although no N-glycoside CKs were determined, the expression pattern of *UGT76C1* (Supplementary Fig. S4D) suggests that *N*-glycosylation did not play a role for leaf-inherent CK inter-conversion under N starvation.

An alternative pathway for limiting the biological activity of CKs is the attachment of ribose or ribose-5′-monophosphate to CK bases (Schmülling, 2004). The release of CK nucleobases from their respective CK ribose 5′-monophosphate is catalysed inter alia in a single-step by cytokinin-specific riboside 5′-monophosphate phosphoribohydrolase encoded by *LONELY GUY* (*LOG*) (Kuroha *et al.*, 2009). Among the four *LOG*s detected in the leaf, particularly *LOG7* (Fig. 6C) and also *LOG4* (Supplementary Fig. S5B) were generally higher expressed under N starvation in the stay-green cvs. NPZ-1 and Apex as compared to their respective early-senescing counterpart cvs. NPZ-2 and Capitol. Particularly LOG7 has been identified as the key enzyme on the whole plant level for the release of highly biological active CKs from their corresponding ribosides (Tokunaga *et al.*, 2012). But despite the cultivar-specific enhanced *LOG7* (and *LOG4*) expression in the stay-green cultivars, the levels of the aCKs were not increased. In contrast to *LOG4* and *LOG7*, *LOG1* expression neither responded to the N supply nor the duration of the treatment (Supplementary Fig. S5A). Although occasionally cultivar differences occurred, no systematic expression pattern existed. However, the generally low change of *LOG1* regulation is most likely the result of the comparable high basal transcript level (ΔC_t_=3.7±0.2). Although *LOG5* showed the highest relative induction with the progression of N starvation-induced leaf senescence (Supplementary Fig. S5C) the initial (DAT0) and final (DAT12) expression levels (ΔC_t_) under both N supplies were comparable low. Thus it is most likely that *LOG5* did not play a decisive role in leaf CK activation. This is in agreement with results in *A. thaliana* where the main sites of *LOG5* transcription were the inflorescences (Kuroha *et al.*, 2009).

The level of biologically active CKs is also determined by their rate of degradation (Schmülling, 2004; Sakakibara, 2006). The CK bases as well as their ribosides are irreversibly degraded by CK-specific oxidase/dehydrogenase (CKX) in a single-step by oxidative cleavage of the side chain (Schmülling *et al.*, 2003). CKX activity is regulated on the transcriptional level, in which significant differences exist between the seven known isoenzymes in the ability to target specific CKs (Schmülling *et al.*, 2003; Galuszka *et al.*, 2007; Kowalska *et al.*, 2010; Gajdošová *et al.*, 2011; Trifunović *et al.*, 2013; Köllmer *et al.*, 2014). The regulation patterns of *CKX1*, *CKX2*, *CKX3*, *CKX6* and *CKX7* during N starvation-induced leaf senescence (Supplementary Fig. S6) suggest that CK degradation most likely did not play a major role for cultivar differences in functional stay-green.

The pool of biologically active CKs in the leaf may be insufficient to fully explain differences in CK-mediated leaf longevity. Of equal or even more importance is the binding of biologically active CKs to the CK-specific receptor histidine kinase AHK3, as well as the subsequent activation of the response regulator ARR2 (Kim *et al.*, 2006). The response regulator ARR2 induces downstream CK-responsive genes and, directly or indirectly, induces or represses a set of target genes responsible for the regulation of leaf senescence (Kim *et al.*, 2006). According to the cultivar differences in N starvation-induced leaf senescence *AHK3* tended to be more highly upregulated in the stay-green cvs. NPZ-1 and Apex as compared to their respective early-senescing counterpart cvs. NPZ-2 and Capitol (Fig. 7A). In accordance to the regulation of *AHK3*, *ARR2* too tended toward higher upregulation in the stay-green cvs. NPZ-1 and Apex as compared to their respective early-senescing counterpart cvs. NPZ-2 and Capitol (Fig. 7B). Thus, the expression patterns suggest that the binding of and the response to biologically active CKs mediated by *AHK3* and *ARR2* are additional leaf-inherent factors responsible for cultivar differences in N starvation-induced leaf senescence.

In conclusion, the present study revealed that cultivar differences in fuctional stay-green under N starvation were primarily governed by leaf-inherent factors. The specific leaf contents of CKs differing in biological activity and the expression of genes involved in CK homeostasis suggest that leaves of early-senescing cultivars were characterized by inactivation of biologically active CKs, whereas in fuctional stay-green cultivar synthesis, activation, binding of and response to biologically active CKs were favoured. Thus, the homeostasis of biologically active CKs was the predominant leaf-inherent factor for cultivar differences in N starvation-induced leaf senescence. The better understanding of the functional stay-green phenotype under N starvation could facilitate the breeding of N-efficient *B. napus* cultivars and thus contribute to reduce the large N balance surplus characteristic of this crop.

## Supplementary data

Supplementary data are available at *JXB* online.

Supplementary Fig. S1. Comparison of cultivars for detaching and N starvation-induced leaf senescence.

Supplementary Fig. S2. SA, JA and ABA in root, the xylem sap and leaf of four cultivars.

Supplementary Fig. S3. Relative Expression of *IPT2*, *IPT5* and *IP9*.

Supplementary Fig. S4. Relative Expression of *UGT73C1, UGT73C4, UGT73C5, UGT76C1* and *UGT85A1*.

Supplementary Fig. S5. Relative Expression of *LOG1, LOG4, LOG5* and *LOG7*.

Supplementary Fig. S6. Relative Expression of *CKX1, CKX2, CKX3, CKX6* and *CKX7*.

Supplementary Fig. S7. N uptake of non-grafted, self-grafted and reciprocally-grafted plants.

Supplementary Fig. S8. N concentrations of non-grafted, self-grafted and reciprocally-grafted plants.

Supplementary Fig. S9. Correlation between specific leaf N content and photosynthesis rate.

Supplementary Table S1. Primer sequences of the genes of interest and the reference gene.

Supplementary Table S2. Statistical comparison of the two grafting experiments.

Supplementary Table S3. F test for the senescence status of the 3rd and 4th leaf.

Supplementary Table S4. F test for salicylic acid, jasmonic acid and abscisic acid.

Supplementary Table S5. F test for cytokinins.

Supplementary Data
