# Supplementary material for: Differences between winter oilseed rape (Brassica napus L.) cultivars in nitrogen starvation-induced leaf senescence are governed by leaf-inherent rather than root-derived signals
Source: J Exp Bot. 2015 May 4;66(13):3669–81. doi: 10.1093/jxb/erv170 (PMC4473979; doi:10.1093/jxb/erv170)
Supplement: Supplementary Data [file supp_66_13_3669__index.html]

Differences between winter oilseed rape (Brassica napus L.) cultivars in nitrogen starvation-induced leaf senescence are governed by leaf-inherent rather than root-derived signals — Supplementary Data 

# Differences between winter oilseed rape (*Brassica napus* L.) cultivars in nitrogen starvation-induced leaf senescence are governed by leaf-inherent rather than root-derived signals

## Supplementary Data

Data files

- Supplementary Data - Supplementary Data
