# Supplementary material for: Differences between winter oilseed rape (Brassica napus L.) cultivars in nitrogen starvation-induced leaf senescence are governed by leaf-inherent rather than root-derived signals
Source: J Exp Bot. 2015 May 4;66(13):3669–81. doi: 10.1093/jxb/erv170 (PMC4473979; doi:10.1093/jxb/erv170)
Supplement: Supplementary Data [file supp_erv170_erv170_SUPPLEMENTARY_DATA_SH.pdf]

Differences between winter oilseed-rape (*Brassica napus* L.) cultivars in nitrogen starvation-induced leaf senescence are governed by leaf-inherent rather than root-derived signals

Fabian Koeslin-Findeklee[1], Martin A. Becker[1], Eric van der Graaff[2], Thomas Roitsch[2,3] and Walter J. Horst[1]

[1]Institute of Plant Nutrition, Leibniz University of Hannover, Herrenhäuser Str. 2, D-30419 Hannover, Germany
[2]Department of Plant and Environmental Sciences, Copenhagen Plant Science Center, University of Copenhagen, Højbakkegård Allé13, DK-2630 Taastrup, Denmark
[3]Global Change Research Centre, CzechGlobe AS CR, v.v.i., Drásov 470, Cz-664 24 Drásov, Czech Republic

Supplementary Data

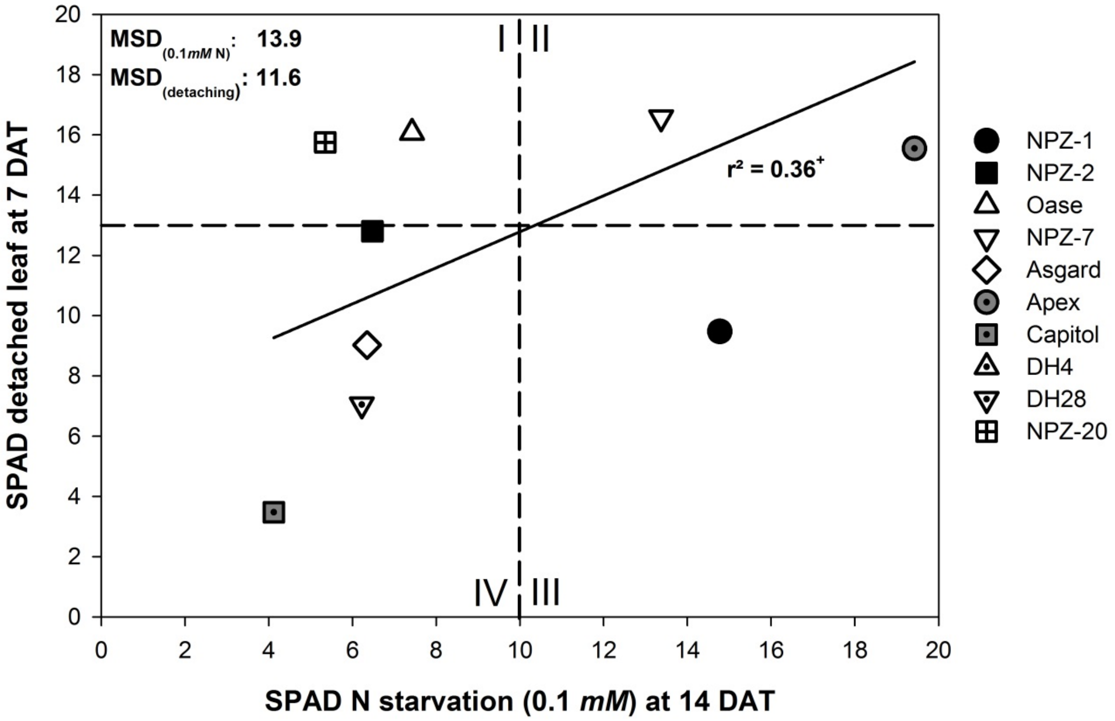

Supplementary Fig. S1. Correlation between SPAD of detached leaves 7 DAT and SPAD of intact N-starved leaves 14 DAT of 10 winter oilseed-rape line-cultivars. The plants were pre-cultured for 28 days at 2.0 *mM* N. Detached leaves were cultured in Erlenmeyer flasks containing deionized water (for experimental details see Koeslin-Findklee et al., 2015). The dashed lines show the average SPAD values across the ten line cultivars. Cultivars in quadrants I and III differ in their responses whereas cultivars in the quadrants II and IV correspond with their responses in SPAD to detaching and N starvation. MSD = Minimum significant difference ($p < 0.05$). For the correlation + indicate significance at $p < 0.10$. Four biological replicates.

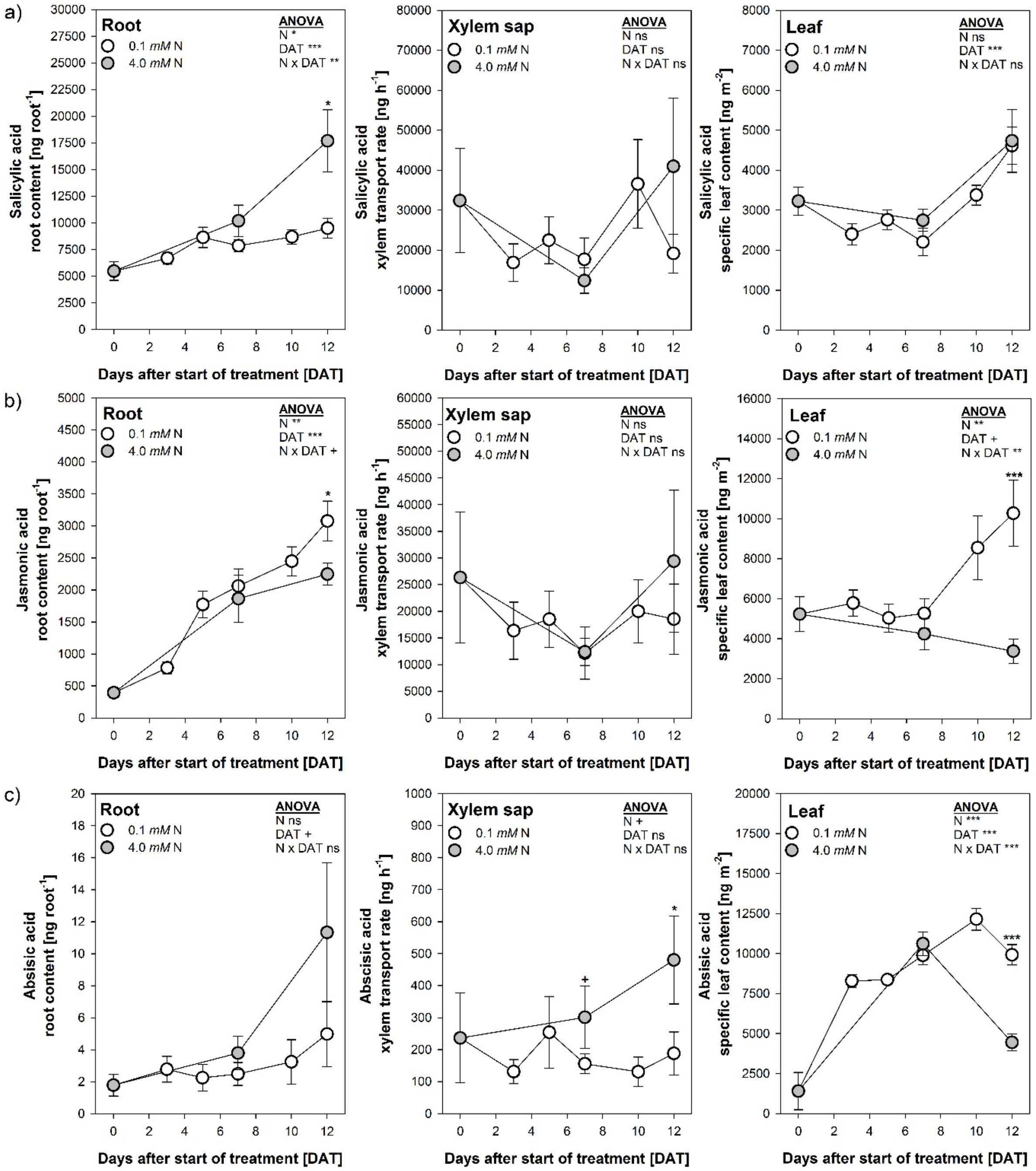

Supplementary Fig. S2. Salicylic acid (a), jasmonic acid (b) and abscisic acid (c) in the root, the xylem sap and the second oldest harvested mature leaf of the winter oilseed-rape cultivars NPZ-1, NPZ-2, Apex and Capitol grown in hydroponics during 12 days N starvation (0.1 *mM*) or optimal N supply (4.0 *mM*). The plants were pre-cultured for 28 days at 2.0 *mM* N. For the ANOVA +, *, **, *** indicate significant differences at p<0.10, <0.05, <0.01, <0.001, respectively. ns, non-significant. At 7 and 12 DAT +, *, *** indicate significant differences between the N supplies at p<0.10, <0.05 <0.001, respectively. The error bars (visible only when greater than the symbols) represent the standard errors of the means across the four cultivars (n=3–4).

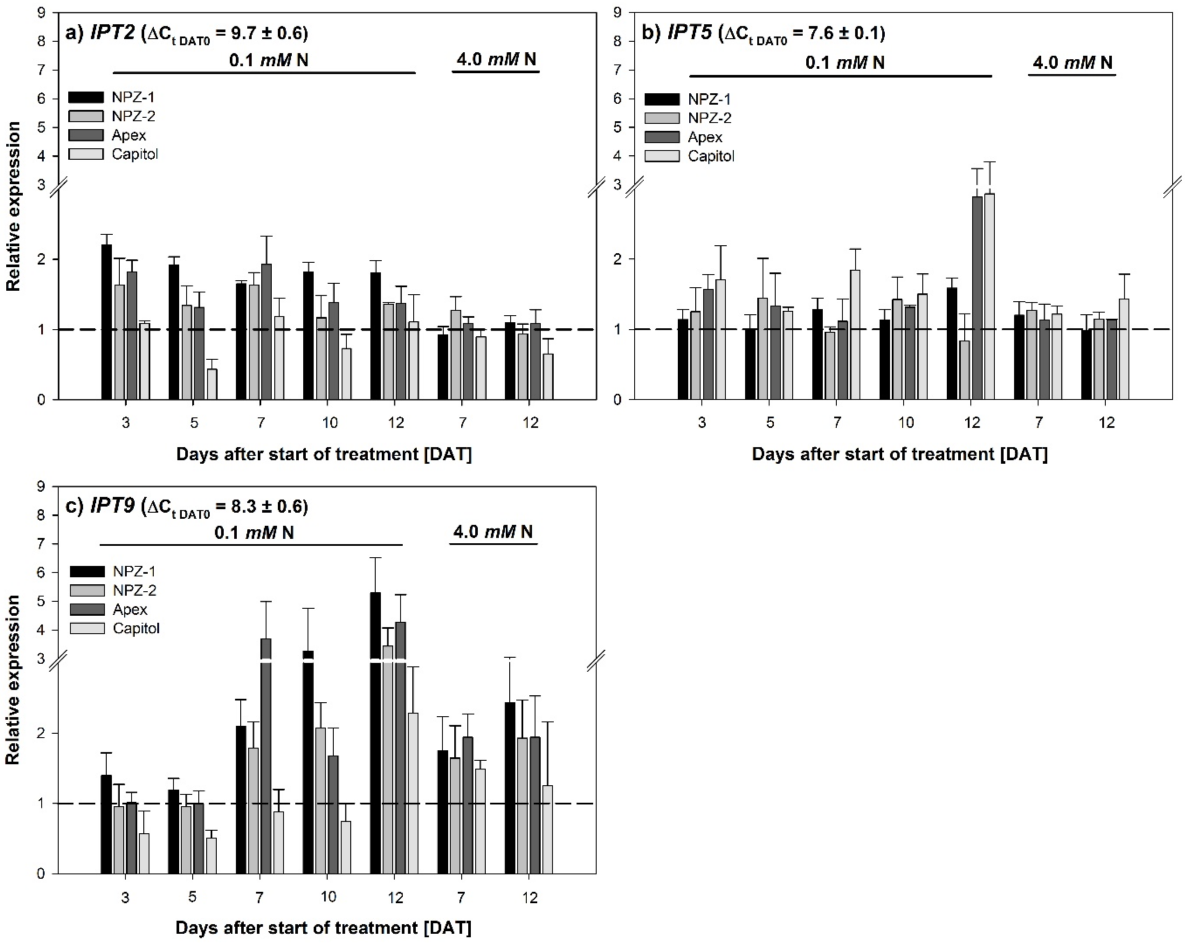

Supplementary Fig. S3. Relative expression ($2^{-\Delta\Delta Ct}$) of the isopentenyltransferase (IPT) genes *IPT2* (a), *IPT5* (b) and *IPT9* (c) in the second oldest harvested mature leaf of four winter oilseed-rape cultivars grown in hydroponics during 12 days N starvation (0.1 *mM*) or optimal N supply (4.0 *mM*). The plants were pre-cultured for 28 days at 2.0 *mM* N. The data are shown relative to the cultivar-specific control at DAT 0 (dashed line). The error bars represent the standard errors of the means (n=3–4). ± indicates the standard deviation of the mean for the ΔC_t across the four cultivars.

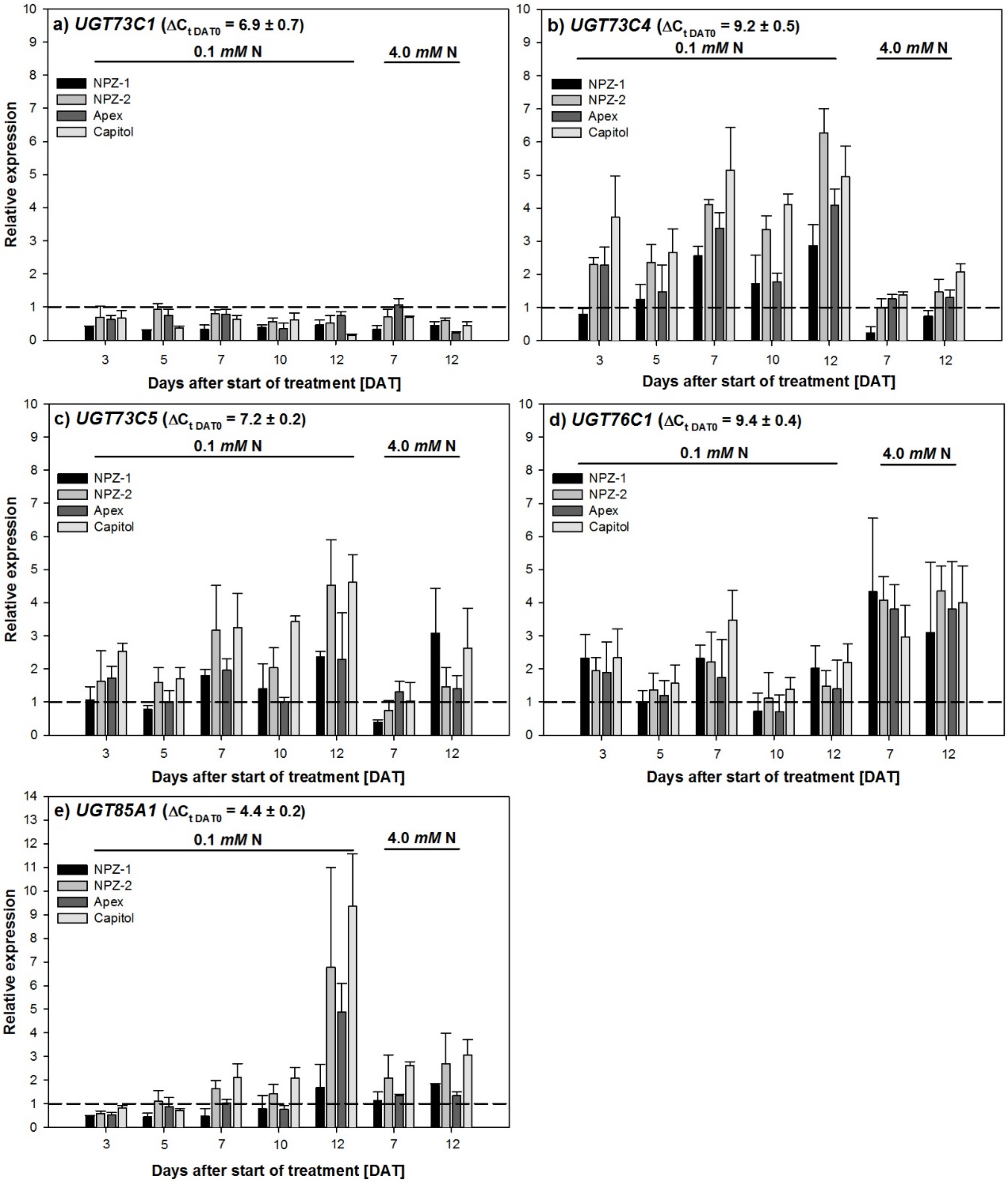

Supplementary Fig. S4. Relative expression ($2^{-\Delta\Delta Ct}$) of the uridine diphosphate glycosyltransferases (UGT) genes *UGT73C1* (a), *UGT73C4* (b), *UGT73C5* (c), *UGT76C1* (d) and *UGT85A1* (e) in the second oldest harvested mature leaf of four winter oilseed-rape cultivars grown in hydroponics during 12 days N starvation (0.1 *mM*) or optimal N supply (4.0 *mM*). The plants were pre-cultured for 28 days at 2.0 *mM* N. The data are shown relative to the cultivar-specific control at DAT 0 (dashed line). The error bars represent the standard errors of the means (n=3–4). ± indicates the standard deviation of the mean for the ΔC_t across the four cultivars.

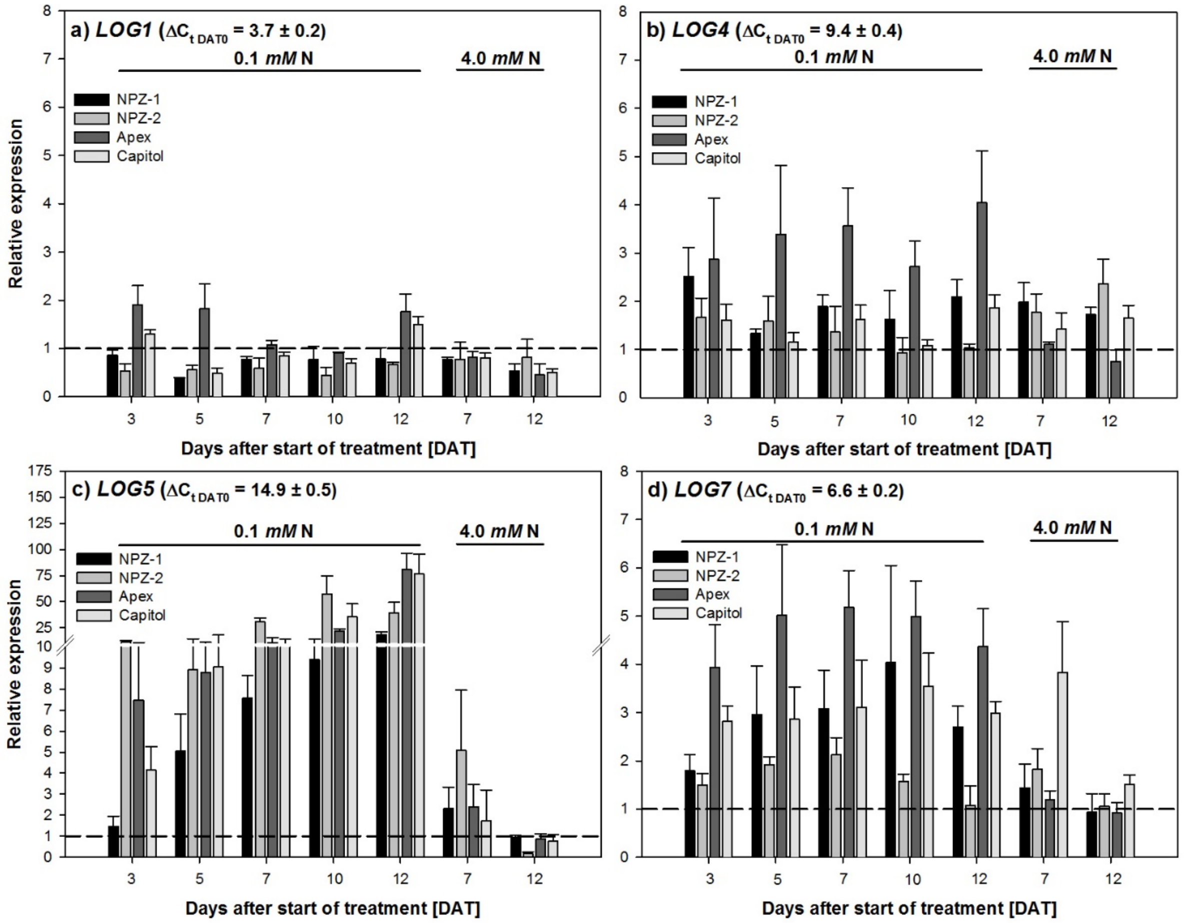

Supplementary Fig. S5. Relative expression ($2^{-\Delta\Delta Ct}$) of the cytokinin ribosid 5′ monophosphate phosphoribohydrolase genes *LOG1* (a), *LOG4* (b), *LOG5* (c) and *LOG7* (d) in the second oldest harvested mature leaf of four winter oilseed rape cultivars grown in hydroponics during 12 days N starvation (0.1 *mM*) or optimal N supply (4.0 *mM*). The plants were pre-cultured for 28 days at 2.0 *mM* N. The data are shown relative to the cultivar-specific control at DAT 0 (dashed line). The error bars represent the standard errors of the means (n=3–4). ± indicates the standard deviation of the mean for the ΔC_t across the four cultivars.

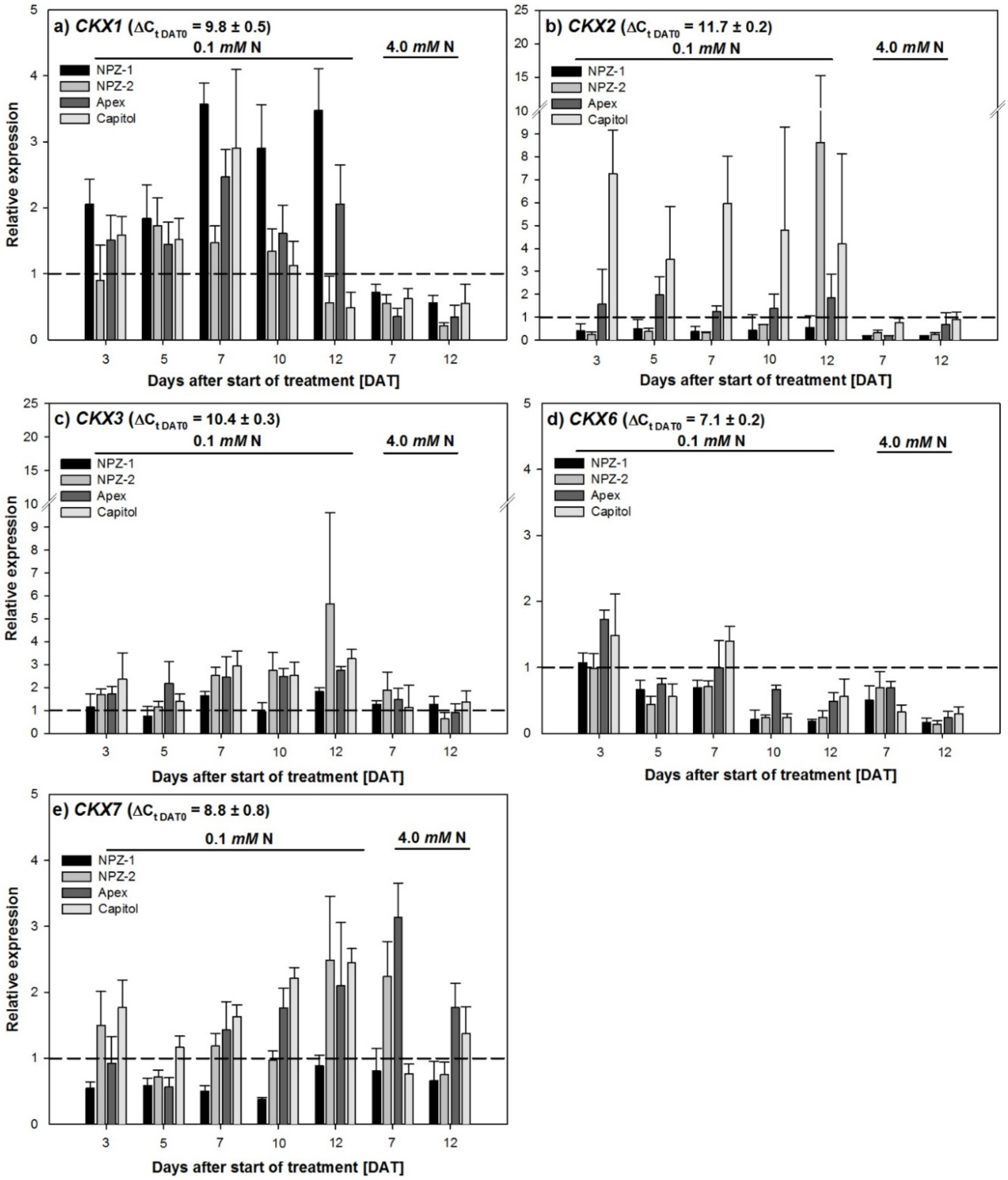

Supplementary Fig. S6. Relative expression ($2^{-\Delta\Delta Ct}$) of the cytokinin oxidase (CKX) genes *CKX1* (a), *CKX2* (b), *CKX3* (c), *CKX6* (d) and *CKX7* (e) in the second oldest harvested mature leaf of four winter oilseed rape cultivars grown in hydroponics during 12 days N starvation (0.1 *mM*) or optimal N supply (4.0 *mM*). The plants were pre-cultured for 28 days at 2.0 *mM* N. The data are shown relative to the cultivar-specific control at DAT 0 (dashed line). The error bars represent the standard errors of the means (n=3–4). ± indicated the standard deviation of the mean for the ΔC_t across the four cultivars.

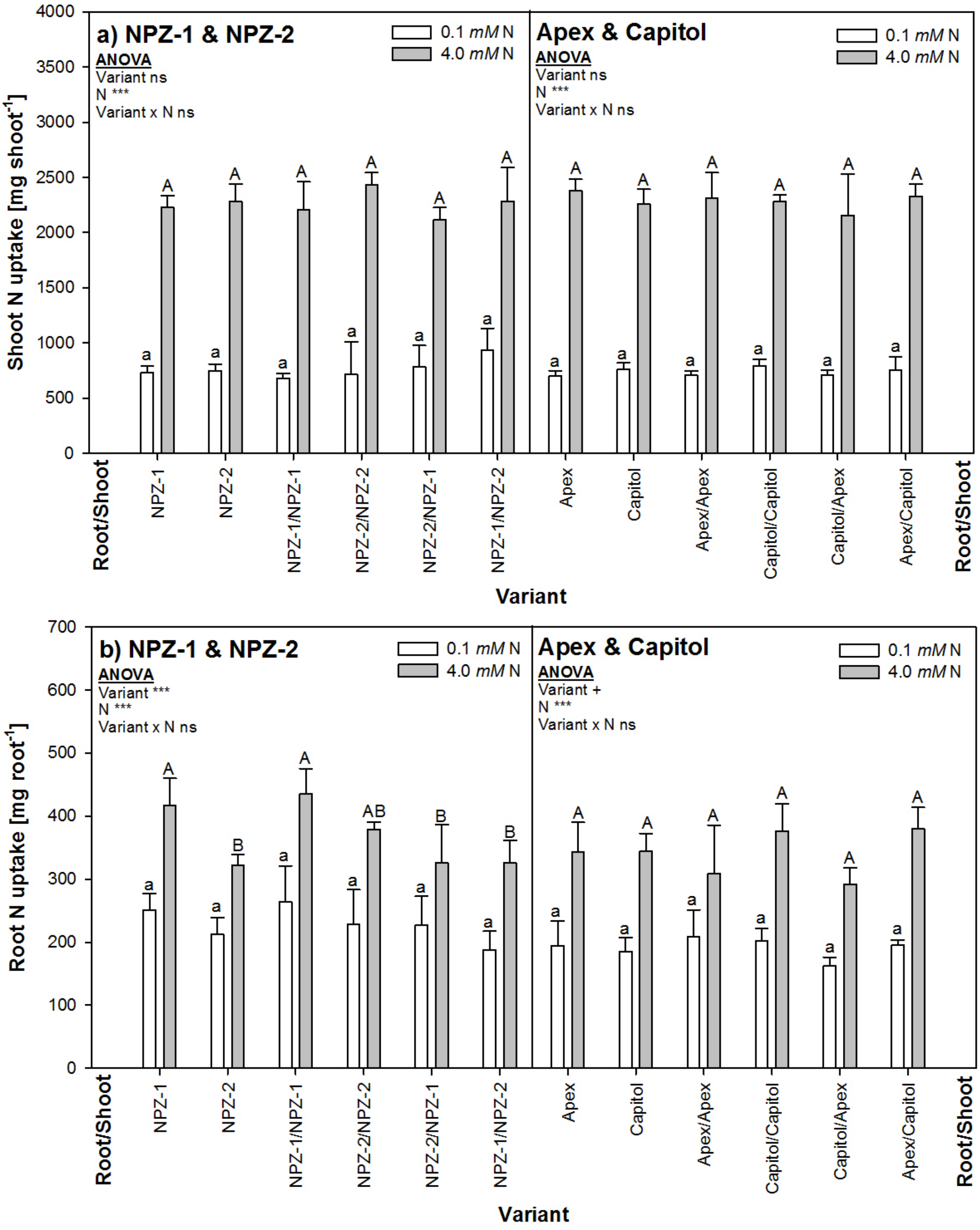

Supplementary Fig. S7. Shoot (a) and root (b) N uptake of non-grafted, self-grafted and reciprocally-grafted plants of the winter oilseed-rape cultivars NPZ-1 & NPZ-2 (left) and Apex & Capitol (right) grown in hydroponics after 12 days of N starvation (0.1 *mM* N) or optimal N supply (4.0 *mM* N). The plants were pre-cultured for 28 days at 2.0 *mM* N. Different letters on top of the columns indicate differences between the variants (p <0.05). For the ANOVA +, *** indicate significant differences at p<0.10, <0.001, respectively. ns, non-significant. The error bars represent

the standard deviations of the means (n=3–4).

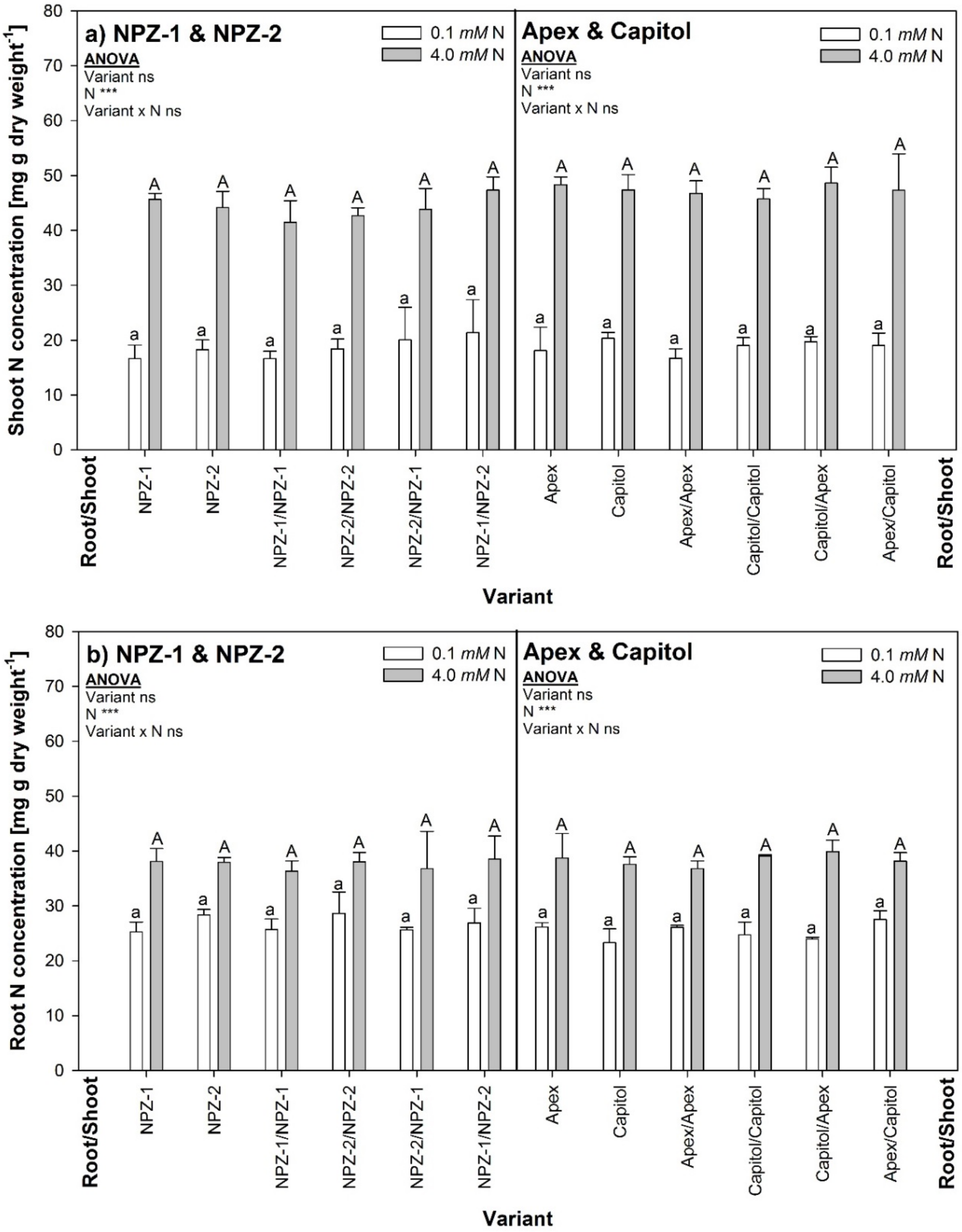

Supplementary Fig. S8. Shoot (a) and root (b) N concentration of non-grafted, self-grafted and reciprocally-grafted plants of the winter oilseed-rape cultivars NPZ-1 & NPZ-2 (left) and Apex & Capitol (right) grown in hydroponics after 12 days of N starvation (0.1 *mM* N) or optimal N supply (4.0 *mM* N). The plants were pre-cultured for 28 days at 2.0 *mM* N. Different letters on top of the columns indicate differences between the variants ($p<0.05$). For the ANOVA *** indicate significant

differences at p<0.001. ns, non-significant. The error bars represent the standard deviations of the means (n=3–4).

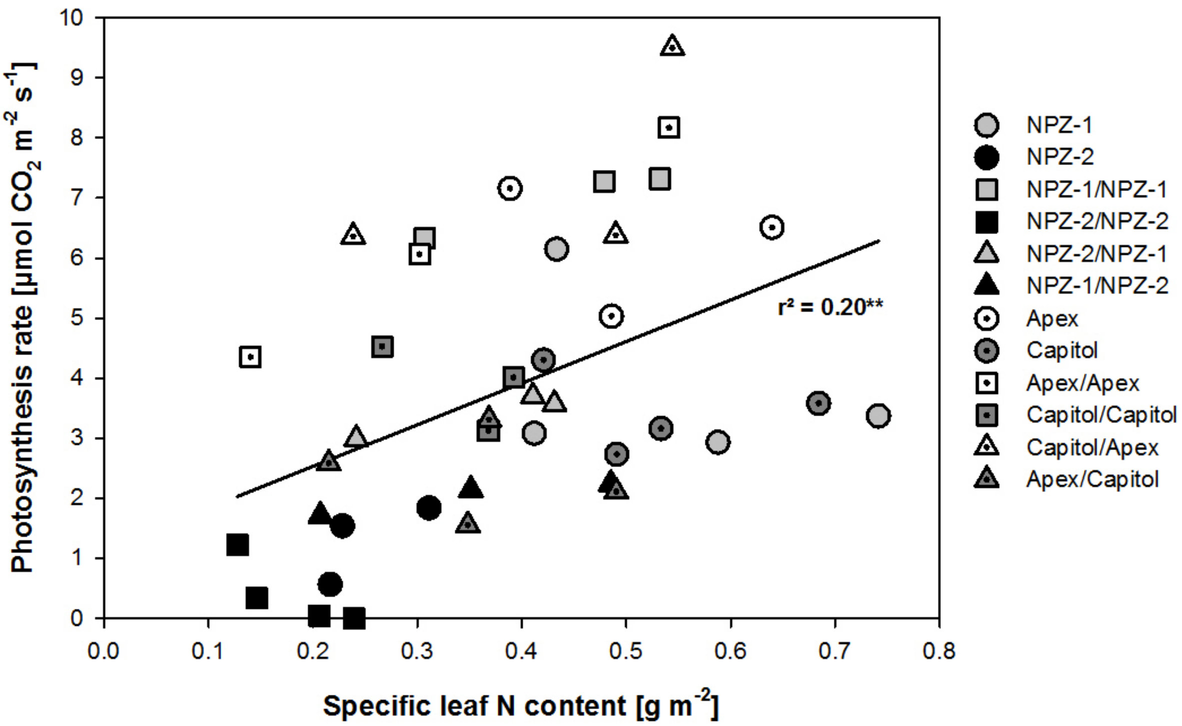

Supplementary Fig. S9. Correlation between specific leaf N content and photosynthesis rate of non-grafted (circles), self-grafted (squares) and reciprocally-grafted (triangles) plants of the winter oilseed-rape cultivars NPZ-1 & NPZ-2 and Apex & Capitol grown in hydroponics after 12 days of N starvation (0.1 *mM* N). The plants were pre-cultured for 28 days at 2.0 *mM* N. For the correlation ** indicate significance at p<0.01.

Supplementary Table S1. F-Test for variant, N supply, leaf position and their interaction on SPAD of the 3rd and 4th leaf (n=3–4) separately for the oilseed-rape cultivar-pairs NPZ-1 & NPZ-2 and Apex & Capitol, respectively. For the ANOVA +, *** indicate significant differences at p <0.10, <0.001, respectively. ns, non-significant.

	**NPZ-1 & NPZ-2**	**Apex & Capitol**
Variant	***	***
N	***	***
Leaf	***	+
Variant x N	***	ns
Variant x Leaf	ns	ns
N x Leaf	ns	ns
Variant x N x Leaf	ns	ns

Supplementary Table S2. Primer sequences of the genes of interest and the reference gene.

Organism	Gene name	Putitative function	Database	Accession number		Oiligo sequence 5' → 3'
B. napus	*EF1-alpha*	Cell elongation	NCBI	DQ312264	+	AGGTCCACCAACCTTGACTG
					-	CCGTTCCAATACCACCAATC
B. napus	*SAG12-1*	Cysteine protease	NCBI	AF089848	+	TACGTGTAGGATGTTGTTGGGCGT
					-	TGGCCATTATGTGCTCAAACGCAG
B. napus	*IPT2*	Cytokinin synthesis	DFCI	TC151192	+	ACGGGATCAGGGAAATCGAAGC
					-	TTATCTCCACCGGGAAGTGAGACG
B. napus	*IPT5*	Cytokinin synthesis	DFCI	EE560823	+	TTCTTTCGAGATGGATGCAAGTGG
					-	AAGAGGGAGGGTTTGGTTACACG
B. napus	*IPT9*	Cytokinin synthesis	DFCI	TC151289	+	TTCCTGTCGAGTCCACGGATTG
					-	AACTCCATTGGCACCTGAGAGC
A. thaliana	*LOG1*	Cytokinin activation	TAIR	AT2G28305	+	ACTCGGAACCGAACTGGTATC
					-	CATTAAACCAATGCTCCCTCCAC
A. thaliana	*LOG4*	Cytokinin activation	TAIR	AT3G53450	+	GTGGTCGCCATGTTATTGGAG
					-	TGCAACTGCTCTTACTTCTCCTAC
A. thaliana	*LOG5*	Cytokinin activation	TAIR	AT4G35190	+	TAATAGCATGGGCACAACTTGG
					-	ATCCACATTTAACAAACCCACAGG
A. thaliana	*LOG7*	Cytokinin activation	TAIR	AT5G06300	+	TCAATTGGGTAACGAGTTGGTG
					-	TTGAGAGACGAGACCCATAAGC
B. napus	*UGT73C1*	Cytokinin inactivation	DFCI	TC139808	+	TATGGAGCATCGGACCGGTTTC
		(reversible)			-	ACCTATGGCCGCCTTATTTCCC
A. thaliana	*UGT73C4*	Cytokinin inactivation?	TAIR	AT2G36770	+	GCGTTCTTGGACGAAATGGTAG
		(reversible?)			-	ATAAGCAGGCTCCAACTCCTG
B. napus	*UGT73C5*	Cytokinin inactivation	DFCI	TC140686	+	GAAGGTGGTTCTGTGCTCTACG
		(reversible)			-	CTGAGACAGAGGAAGATTGCAGAC
B. napus	*UGT76C1*	Cytokinin inactivation	DFCI	DY007790	+	AACAACTGCGAGAACCCGCTAC
		(irreversible)			-	TCTCACCACCTTCCTCTGTTTCTG
B. napus	*UGT85A1*	Cytokinin inactivation	DFCI	TC139953	+	GTTTGCTGGTCGGCAAAGAACG
		(reversible)			-	TCGACGTTGGAGAGTCTCTGTG
B. napus	*CKX1*	Cytokinin degradation	DFCI	EV217277	+	TGATGTCCACAACGCGTCCAAG
					-	TGGATGGAGAATTGCCAGAGGTG
B. napus	*CKX2*	Cytokinin degradation	DFCI	TC153766	+	TGTCGACGATGCCGTTTGAC
					-	ACAATGACACTGGAGTTGACTACG
A. thaliana	*CKX3*	Cytokinin degradation	TAIR	AT5G56970	+	CATCGTGTCATCTACTGCCTTG
					-	CGCTTAACTCCTCCATTTCCTC
A. thaliana	*CKX6*	Cytokinin degradation	TAIR	AT1G75450	+	CTGTCCAATGCTGGAATAAGCG
					-	TCCTGTGACAATCTCCAGTTGATG
B. napus	*CKX7*	Cytokinin degradation	DFCI	TC140449	+	ATATCGCCGGGAAGGACTTTGG
					-	ATATCTTCCGGTCCTAGCGGTCTC
A. thaliana	*AHK3*	Cytokinin receptor	TAIR	AT1G27320	+	GCAGATGTTGCAAAGTCACAGTTC
					-	TGCCTGTGCGGTCCTAACATAATC
A. thaliana	*ARR2*	Cytokinin response	TAIR	AT4G16110	+	AAAGAGTGGCGGAGACAGTGAC
		regulator			-	GGATGGGATGCCTTCCTGTTTG

Supplementary Table S3. Statistical comparison of the two grafting experiments for the factors variant, experiment and their interaction for SPAD 12 days after exposure to N starvation. For the ANOVA +, *, *** indicate significant differences at p<0.10, <0.05, <0.001, respectively. ns, non-significant. The ANOVA (p<0.05) is based on the ranked values of the biological replicates (n=3–4).

	**Apex & Capitol**		**NPZ-1 & NPZ-2**	
	**N [*mM*]**			
	**0.1**	**4.0**	**0.1**	**4.0**
Variant	***	+	***	ns
Experiment	ns	ns	ns	ns
Variant x Experiment	ns	ns	*	ns

Supplementary Table S4. F-Test for cultivar, N supply, DAT and their interaction on the contents or concentrations of salicylic acid, jasmonic acid and abscisic acid in the root, xylem sap and leaf tissue of the second and third oldest harvested leaves of the winter oilseed-rape cultivars NPZ-1, NPZ-2, Apex and Capitol (n=3–4). For the ANOVA +, *, **, *** indicate significant differences at p<0.10, <0.05, <0.01, <0.001, respectively. ns, non-significant; ---, not applicable.

	**Salicylic acid**			**Jasmonic acid**			**Abscisic acid**		
	**Root content [ng root^{-1}]**	**Xylem transport [ng h^{-1}]**	**Leaf content [ng m^{-2}]**	**Root content [ng root^{-1}]**	**Xylem transport [ng h^{-1}]**	**Leaf content [ng m^{-2}]**	**Root content [ng root^{-1}]**	**Xylem transport [ng h^{-1}]**	**Leaf content [ng m^{-2}]**
Cultivar	***	ns	***	***	ns	ns	+	ns	+
N	**	ns	ns	**	ns	**	+	+	***
DAT	***	ns	***	***	ns	*	***	ns	***
Leaf	---	---	***	---	---	---	---	---	ns
Cultivar x N	ns	ns	ns	ns	ns	ns	ns	ns	***
Cultivar x DAT	+	ns	***	ns	ns	ns	**	ns	***
Cultivar x Leaf	---	---	***	---	---	---	---	---	ns
N x DAT	***	ns	*	ns	ns	**	ns	ns	***
N x Leaf	---	---	*	---	---	---	---	---	*
Leaf x DAT	---	---	***	---	---	ns	---	---	***
Cultivar x N x DAT	*	ns	*	ns	ns	+	ns	ns	***
Cultivar x N x Leaf	---	---	ns	---	---	ns	---	---	**
Cultivar x N x DAT x Leaf	---	---	***	---	---	ns	---	---	***
N x DAT x Leaf	---	---	+	---	---	*	---	---	ns

Supplementary Table S5. F-test for cultivar, N supply and duration of N starvation (DAT) on the contents or concentrations of cytokinins in root, xylem sap and leaf tissue of the second and third oldest harvested leaves of the winter oilseed-rape cultivars NPZ-1, NPZ-2, Apex and Capitol (n=3–4). For the ANOVA +, *, **, *** indicate significant differences at p<0.10, <0.05, <0.01, <0.001, respectively. ns, non-significant; ---, not applicable.

	**Cytokinins**					
	**Root content [ng root^{-1}]**		**Xylem transport [ng h^{-1}]**		**Leaf content [ng m^{-2}]**	
	**Active**	**Activatable**	**Active**	**Activatable**	**Active**	**Activatable**
Cultivar	ns	*	ns	*	ns	***
DAT	***	***	ns	ns	+	***
N	ns	**	ns	*	ns	***
Leaf	---	---	---	---	ns	ns
Compound	***	***	***	***	***	***
Cultivar x DAT	ns	ns	ns	***	ns	*
Cultivar x N	ns	ns	ns	+	ns	*
Cultivar x Leaf	---	---	---	---	ns	ns
Cultivar x Compound	ns	*	ns	ns	ns	***
Cultivar x DAT x N	ns	ns	+	**	ns	ns
Cultivar x DAT x Leaf	---	---	---	---	ns	ns
Cultivar x DAT x Compound	ns	ns	ns	**	ns	*
Cultivar x DAT x N x Leaf	---	---	---	---	ns	ns
Cultivar x DAT x N x Compound	ns	ns	ns	**	ns	ns
Cultivar x DAT x N x Leaf x Compound	---	---	---	---	ns	ns
DAT x N	ns	***	ns	ns	ns	**
DAT x Leaf	---	---	---	---	ns	ns
DAT x Compound	***	***	ns	ns	ns	***
DAT x N x Leaf	---	---	---	---	ns	ns
DAT x N x Compound	ns	***	ns	ns	ns	**
DAT x N x Leaf x Compound	---	---	---	---	ns	ns
N x Leaf	---	---	---	---	ns	ns
N x Compound	*	**	ns	+	*	***
N x Leaf x Compound	---	---	---	---	ns	ns
Leaf x Compound	---	---	---	---	ns	ns
